# Complete Columbian mammoth mitogenome suggests interbreeding with woolly mammoths

**DOI:** 10.1186/gb-2011-12-5-r51

**Published:** 2011-05-31

**Authors:** Jacob Enk, Alison Devault, Regis Debruyne, Christine E King, Todd Treangen, Dennis O'Rourke, Steven L Salzberg, Daniel Fisher, Ross MacPhee, Hendrik Poinar

**Affiliations:** 1 McMaster Ancient DNA Centre, Department of Anthropology, McMaster University, 1280 Main Street West, Hamilton, Ontario L8S 4L9, Canada; 2 Muséum national d'Histoire naturelle, UMR 7206 Eco-anthropologie, Equipe "génétique des populations humaines," 57 rue Cuvier, CP139, 75231 Paris Cedex 05, France; 3 Center for Bioinformatics and Computational Biology, 3115 Biomolecular Sciences Bldg #296, University of Maryland, College Park, MD 20742, USA; 4 Department of Anthropology, University of Utah, 270 S. 1400 East Room 102, Salt Lake City, UT 84112-0060, USA; 5 Museum of Paleontology and Department of Geological Sciences, University of Michigan, 1109 Geddes Ave, Ann Arbor, MI 48109-1079, USA; 6 Division of Vertebrate Zoology, American Museum of Natural History, Central Park West @ 79th St, New York, NY 10024, USA

## Abstract

**Background:**

Late Pleistocene North America hosted at least two divergent and ecologically distinct species of mammoth: the periglacial woolly mammoth (*Mammuthus primigenius*) and the subglacial Columbian mammoth (*Mammuthus columbi*). To date, mammoth genetic research has been entirely restricted to woolly mammoths, rendering their genetic evolution difficult to contextualize within broader Pleistocene paleoecology and biogeography. Here, we take an interspecific approach to clarifying mammoth phylogeny by targeting Columbian mammoth remains for mitogenomic sequencing.

**Results:**

We sequenced the first complete mitochondrial genome of a classic Columbian mammoth, as well as the first complete mitochondrial genome of a North American woolly mammoth. Somewhat contrary to conventional paleontological models, which posit that the two species were highly divergent, the *M. columbi *mitogenome we obtained falls securely within a subclade of endemic North American *M. primigenius*.

**Conclusions:**

Though limited, our data suggest that the two species interbred at some point in their evolutionary histories. One potential explanation is that woolly mammoth haplotypes entered Columbian mammoth populations via introgression at subglacial ecotones, a scenario with compelling parallels in extant elephants and consistent with certain regional paleontological observations. This highlights the need for multi-genomic data to sufficiently characterize mammoth evolutionary history. Our results demonstrate that the use of next-generation sequencing technologies holds promise in obtaining such data, even from non-cave, non-permafrost Pleistocene depositional contexts.

## Background

Conventional paleontological models [1-4] of North American mammoth evolution posit that at least two species occupied the continent during the late Pleistocene (150,000 to 10,000 years ago: *Mammuthus primigenius *(woolly mammoths (WMs)) evolved in Eurasia and immigrated to North America in the late Pleistocene, whereas *Mammuthus columbi *(Columbian mammoths (CMs)) evolved locally from an earlier Pleistocene immigrant ancestor (*Mammuthus meridionalis *[1,2] or *Mammuthus trogontherii *[3,4]). The species are morphologically differentiated by physical size (CMs were some 25% taller than WMs [5]), molar complexity (CMs displayed more 'primitive' crown height and lamellar configuration), and skull morphology (CMs possessed a more downturned mandibular symphysis and more laterally oriented tusk alveoli) [1,5]. Some of these traits are considered adaptations to their disparate habitats: WMs inhabited cold and arid periglacial regions, while CMs inhabited the temperate regions of the southern latitudes. Continental populations of both species went extinct during the Pleistocene-Holocene transition some 10,000 years ago.

**Figure 1:**
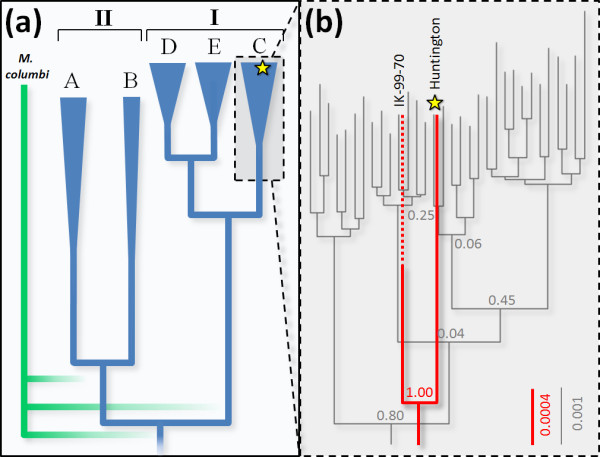
**Mammoth mitochondrial DNA cladograms**. **(a) **WM lineages (blue) are summarized from previous studies [9-11] with clades indicated and haplogroups labeled at the tips. Hypothetical CM lineage positions (green) are expected positions derived from strict interpretations of paleontological models that posit the two species were separate since the early Pleistocene. The multiple node positions reflect the general uncertainty surrounding the chronology and identity of the WM lineage common ancestor. The position of WM haplogroup B is poorly resolved, exhibiting deep common ancestry with the other haplogroups. Haplogroups A and C are endemic to Eurasia and North America, respectively; haplogroups B, D, and E occur on both continents. Radiocarbon chronologies indicate that haplogroup A went extinct approximately 35,000 ^14^Cya, and clade I by approximately 3,200 ^14^Cya. Calculated MRCA ages for all nodes yield wide confidence intervals. **(b) **Our estimated mtDNA cladograms of haplogroup C are depicted using two datasets: the black cladogram and associated scale and posterior probabilities (parameter set 1b, Figure S4 in Additional file 3) are estimated from 743 bp for which several dozen mammoths have been sequenced, whereas the red cladogram and associated scale and posterior probabilities (parameter set 4b, Figure S8 in Additional file 3) are estimated from full mitochondrial genomes, for which only one other haplogroup C mammoth has been sequenced. Each tip in the black cladogram represents a haplotype. *M. columbi *(haplotype C32) as represented by the Huntington Mammoth is indicated with a yellow star. Scale units are substitutions per site.

Recent paleontological reconsiderations [6-8] and mitochondrial DNA (mtDNA) phylogeographic studies of predominantly Beringian mammoths [9-13] reveal a complex evolutionary history (Figure 1a). Their populations harbored diverse genetic lineages, two of which, haplogroups A and C, were endemic to Eurasia and North America, respectively. Certain population dynamics - including major immigration/replacement events and regional genetic introgression - have been offered as explanations for this complexity [10,11], but its precise origins have proven difficult to define within the broader context of Pleistocene biogeography and paleoecology. This is the case for at least two reasons: first, key coalescent dates remain difficult to measure, in large part due to lack of sequence breadth and methodological shortcomings [14,15]; and second, almost nothing is known about the mtDNA phylogeny of *Mammuthus *beyond Beringian late Pleistocene mammoths (and thus probably exclusively *M. primigenius*). One potential solution to both problems - and means to hone conceptions of Pleistocene mammoth evolution in general - is to sequence DNA from one or more closely related but distinct mammoth species and use it as a temporal and taxonomic calibration tool within the mammoth gene tree. Owing to their apparently separate evolutionary history (Figure 1a) and reasonably well-dated recent divergence from WMs about 1 to 2 million years ago [16], CMs are excellent candidates for this role. To this end we targeted CM remains for mitogenomic sequencing.

We selected the Huntington Mammoth [17] for this purpose on account of its secure morphological identification, direct radiocarbon date (11,220 ± 110 ^14^Cya (radiocarbon years ago), exceptional biomolecular preservation [18] and geographic provenience (Fairview, UT, USA), far south of the Wisconsinan glaciers. Typical strategies for DNA sequencing of paleontological specimens would employ a pre-sequencing targeted enrichment approach, through the use of either labor-intensive PCR or hybridization techniques [19]. However, following serial extraction and library preparation of our specimen, a quantitative PCR-based metric projected a sufficient ratio of target to non-target DNA to warrant a shotgun-based metagenomic sequencing approach, for which we employed the Illumina platform (see Materials and methods; Additional file 1).

## Results and Discussion

Of the over 27 million reads longer than 50 bp obtained from the Huntington sample library, between 6,000 and 9,000 (0.02 to 0.03%) mapped to a WM reference mitogenome [GenBank: NC007596.2] [20] depending on software assembly parameters (Table S3 in Additional file 2). This provided an average unique read depth of approximately 23 × for the entire mitochondrial genome, excluding the VNTR region (positions 16,157 to 16,476). Roughly 2 million reads also mapped to the African elephant (*Loxodonta africana*) nuclear genome, providing approximately 0.03 × coverage of the entire nuclear genome of the animal, and bringing the total likely mammoth DNA read count to approximately 7% of all sequences. Such a proportion of total endogenous DNA is consistent with taphonomic models for DNA preservation in temperate burial contexts [21], as well as experimental data from other non-permafrost remains [22,23]. The coverage depth ratio we observe between mitochondrial and nuclear reads (approximately 800 ×) also falls within the range estimated in other mammoth specimens (245 to 17,000 × [24]). This low nuclear read coverage depth also lends evidence that potential Numts make no significant contribution to the consensus generated from the mitochondrial assembly.

To ensure the authenticity of the mitogenome sequence, we amplified, cloned and sequenced PCR products of WM haplotype-defining regions of the cytochrome *b *gene and hypervariable region from multiple extractions of the Huntington mammoth in two separate ancient DNA facilities. These all yielded consensus sequences 100% identical to the shotgun consensus where they overlapped. Furthermore, we sequenced the same loci from PCRs of another securely identified *M. columbi *(the Union Pacific mammoth, University of Wyoming 6368, found near Rawlins, WY, USA [25,26]), which yielded identical sequences to those acquired for Huntington. Finally, to control for ascertainment bias in assembly of the whole mitogenome, we mapped the Illumina sequencing reads to an Asiatic elephant (*Elephas maximus*) mitogenome [GenBank: DQ316068] and obtained a 99.98% identical consensus sequence where it overlapped with the WM assembly consensus. Thus, we are confident that the final Huntington mammoth mitogenome sequence derives from the genuine endogenous mtDNA of the animal.

Bayesian phylogenetic analysis demonstrates that the Huntington mammoth mitogenome is largely indiscernible from those of endemic North American WMs (Figure 1b). For all model and parameter variants (Table S7 in Additional file 2, Figures S3, S4, S5, S6, S7, and S8 in Additional file 3), the sequence sorts securely within haplogroup C, a subclade additionally represented by dozens of WMs from Alaska and the Yukon [11]. To test for this relationship at the entire mitogenomic level, we also sequenced the first complete mitogenome of a WM from this haplogroup (IK-99-70, from the Alaskan North Slope, USA), which confirmed Huntington's phylogenetic position within haplogroup C (Figure 1b).

At first glance, these results would suggest that, contrary to a strict interpretation of traditional paleontological models for their evolution, CMs and WMs did not descend from populations that were wholly separate since the early Pleistocene. One interpretation could be that mitochondrial haplogroup C corresponds to descendants of immigrant mammoth populations that ultimately gave rise to *M. columbi*. But without expansion, this interpretation would fail to explain why haplogroup C belongs to mammoths with both CM and WM morphologies. Indeed, certain paleontological interpretations have already suggested that CMs and WMs were more closely related than typically thought, even 'geoclinal or chronoclinal variants' [27] descending from a very recent common ancestor. We find that our results also warrant consideration of an alternative scheme, one that operates within existing paleontological models but that accommodates incomplete reproductive barriers between CMs and WMs during some period(s) of their evolutionary history.

mtDNA phylogenies are often inconsistent with species phylogenies [28], especially for populations with sex-biased dispersion and breeding patterns. This is particularly true for extant elephants [29,30], which exhibit male-mediated gene flow between matriarchal herds, rendering their mtDNA phylogenies incomplete representations of breeding history. For example, Asiatic elephant and WM populations both harbor(ed) at least two highly divergent mitochondrial lineages without corresponding morphological differentiation [9-11,31]. Between CMs and WMs, we observe the opposite situation, where their morphological distinction appears to have little mitochondrial genetic correlation. One potential explanation for this is that incomplete lineage sorting (ILS) resulted in the maintenance in CM populations of what ultimately became more WM-like mitochondrial lineages. However, if this were the case, we would expect the CM-WM most recent common ancestor (MRCA) to be positioned much deeper in the cytochrome *b*/hypervariable region phylogeny than observed. Our and previous [11] dual-calibrated estimates for the MRCA for the entirety of haplogroup C dates to the middle Pleistocene (Table S7 in Additional file 2), with the CM-WM MRCA necessarily occurring much more recently, long after their purported species divergence. That said, the haplogroup C full mitochondrial dataset is too small to completely rule out ILS during CM-WM speciation as a plausible explanation.

**Figure 2:**
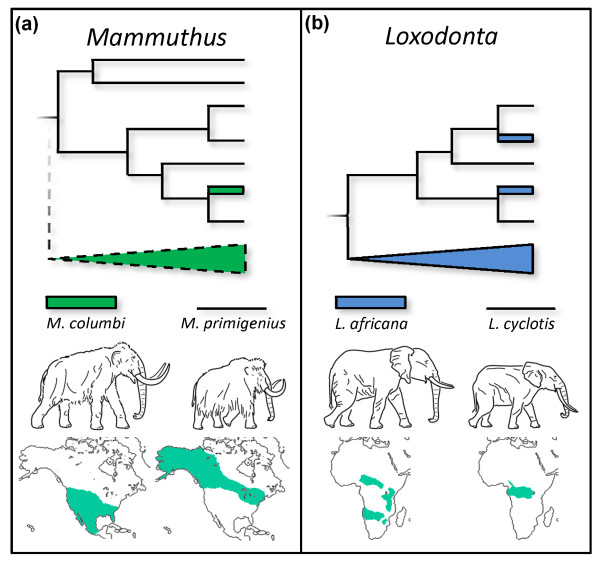
**Schematic representation of elephantid mtDNA phylogenies under introgression scenarios**. **(a,b) **Hypothetical mammoth (b) (this study) and observed African elephant (a) [38] cladograms, with male body size comparisons and predominant geographic ranges of the species indicated. Solid lines represent observed data; dashed lines represent predicted but presently unobserved lineages under an *M. primigenius*-*M. columbi *introgression hypothesis.

At present, however, we suspect that hybridization between CMs and WMs may be a more parsimonious explanation for our observations. Under one conception, haplogroup C could have been a predominantly CM haplogroup that introgressed into WM populations, at such a frequency that it came to dominate the North American mitochondrial gene pool of that species. The fact that both CMs sequenced here are haplogroup C would lend some support to this hypothesis. Another possibility is that introgression occurred in the opposite direction, such that WM-typical haplogroup C introgressed into CM populations (Figure 2a). From a behavioral perspective, this configuration is perhaps more likely, especially in light of phenomena documented in extant African forest (*Loxodonta cyclotis*) and savanna (*L. africana*) elephants (Figure 2b). These living species are morphologically distinct and deeply divergent at many nuclear loci [32-35], but are known to interbreed at forest-savanna ecotones [36,37]. The result is 'cytonuclear dissociation' [38] between genomes in hybrid individuals, such that forest-typical mitochondrial haplotypes occur at low frequency in savanna populations. Hypothetically, this is driven by savanna males reproductively out-competing physically smaller forest males [38], producing unidirectional backcrossing of hybrid females into savanna populations. Since mammoths were probably very similar to modern elephants in social and reproductive behavior [4,27], it is conceivable that WMs and the physically larger CMs engaged in a similar dynamic when they encountered each other. Indeed, hybridization between CMs and WMs has already been suggested by others [39], and genetic exchange may explain mammoths bearing CM-WM intermediate morphologies. Such mammoths are frequently found in areas where CMs and WMs overlapped in time and space, such as the Great Lakes region [2]. Some of these apparent intermediates have been formally named (for example, *Mammuthus jeffersonii*), but their taxonomic identity is questionable. Indeed, the large number of synonyms currently registered for North American mammoths [40] is at least partly a function of efforts by earlier systematists to come to grips with the large amount of morphological variation expressed within *Mammuthus *(or *Elephas*). Although the Huntington mammoth exhibits no such morphological intermediacy and was found quite distant from the documented WM range, its status as a genetic hybrid would not be inconsistent with the modern analog: forest haplotype-bearing savanna elephants can be found several thousands of kilometers from modern ecotones, bearing no phenotypic indication of hybridism [38].

Both the ILS and introgression hypotheses discussed above provide straightforward testable predictions. First, under a WM-CM introgression scenario, some presently unidentified and distinct mitochondrial haplogroup should characterize a significant percentage of CM lineages, rendering their mitogenomes polyphyletic, as they are in *L. africana *(Figure 2). While we also observe a likely C haplotype in short sequences from one other well-identified terminal Pleistocene *M. columbi*, only a broad population-level survey of CM genetic diversity can rigorously test this prediction. Second, under the introgression hypothesis, CMs with WM-type mitogenomes should possess nuclear genes that are significantly more divergent from WMs than all haplogroup C mammoths are from each other. On the other hand, an ILS scenario would predict that CM and WM nuclear genes should show a similar degree of divergence as is detected between haplogroup C mitogenomes. Though we did recover several million nuclear sequences from the Huntington DNA library, the very low coverage depth provided by these reads is not sufficient for reliable nuclear divergence estimates between CMs and WMs. However, we anticipate that targeted enrichment techniques [41,42] prior to high-throughput sequencing will provide the necessary coverage depth to test these hypotheses in the near future.

## Conclusions

The revealed mitochondrial phylogenetic position of *M. columbi *does not immediately clarify complexities and chronological uncertainties previously observed in mammoth mtDNA phylogeny. Instead, it emphasizes that the unique reproductive behavior of elephantids necessitates a multi-genomic approach to characterizing their evolutionary history, as has been so effectively used in studies of living elephants. Their very recent mitochondrial common ancestry strongly suggests that CMs and WMs interbred at some point, most likely post-dating their morphological divergence, and in a fashion that confounds simple correlation of mtDNA phylogeny to evolutionary models derived from mammoth morphology alone. However, the precise mode and setting of genetic interchange between WMs and CMs are elusive, and therefore all hypotheses explaining our observations warrant testing. The possibility that hybridization explains our data is particularly tantalizing, since in many animals, interspecific hybridization accompanies population displacement and/or expansion resulting from habitat reconfiguration [43,44]. Thus, interbreeding between extinct late Pleistocene taxa - especially keystone herbivores like mammoths - could serve as an indicator of major ecological events, including those surrounding the megafaunal extinctions. Our results demonstrate that the use of next-generation sequencing technologies holds promise in rigorously testing such hypotheses using full ancient genomic data, even from non-cave, non-permafrost Pleistocene depositional contexts.

## Materials and methods

### Samples

We included two *M. columbi *(Columbian mammoths) and one *Mammuthus *sp. in the sample set, stored at room temperature at our laboratory.

#### Huntington mammoth - *M. columbi*

College of Eastern Utah Museum CEUM897 is associated with numerous radiocarbon dates, though 11,220 ± 110 ^14^Cya is probably most accurate [17]. It was discovered in 1988 during excavation of a stream for dam construction, at the southeast end of what is now Huntington Reservoir, just east of Fairview, Utah, USA. This 60+ year old bull is exceptionally well preserved, and exhibits the classic character suite of his species, including low molar lamellar frequency (Figure S1 in Additional file 3), broadly divergent tusk alveoli, a markedly downturned mandibular symphysis, and tremendous body size. We used tusk fragments for the shotgun sequencing, and both tusk and bone samples for PCR and Sanger sequencing.

#### Union Pacific mammoth - *M. columbi*

University of Wyoming UW6368 is dated to 11,280 ± 350 ^14^Cya [25,26]. It was discovered in 1960 by a gas well-drilling crew while drag-lining a spring site southwest of Rawlins, Wyoming, USA. Fragments of molar teeth were used for PCR and Sanger sequencing.

#### MPC IK-99-70 - *Mammuthus *sp

Specimen found in the Upper Ikpikpuk River (70° 47'N, 154° 25'W) on the Alaskan North Slope of the USA. Provenience strongly suggests that it is *M. primigenius*. Radiocarbon dated to 41,510 ± 480 ^14^Cya (Beta #264909, Beta Analytic Inc., Miami, FL, USA). The mitochondrial hypervariable region for this specimen was partially sequenced previously [11] and falls within haplogroup C (haplotype C30). Its exceptional DNA preservation prompted its use in the multiplex experiments.

### Sequence acquisition, assembly, and classification

Procedures were performed at a number of laboratories [45-49]. Detailed descriptions of wet laboratory procedures used for sequence acquisition, as well as laboratory procedures for data assembly, can be found in Additional file 1. Primers were taken from previous publications or newly designed using the Integrated DNA Technologies SciTools OligoAnalyzer 3.1 [50]. Pre-sequencing preservation evaluations were performed following [24,51,52]. We used a metagenomic high-throughput sequencing approach to characterize the whole mitochondrial genome of the Huntington mammoth, and multiplex PCR combined with high-throughput sequencing to obtain the whole mitochondrial genome of IK-99-70. We also cloned and sequenced several PCR products from the mammoths, with independent PCR amplification, cloning and sequencing of products from the Huntington mammoth performed at a separate laboratory. We assembled mitochondrial reads with AMOScmp [53] using NUCmer [54] as well as with Geneious 5.1.7 [55] and then visualized assemblies using amosvalidate [56], Hawkeye [57] and Geneious. The Huntington nuclear genome read assemblies were built using these and also classified using PhymmBL [58], comparing previously published WM nuclear genome sequences [59] and the *L. africana *nuclear genome sequence [60]. Sequence read files for Huntington and IK-99-70 are deposited in the NCBI Short Read Archive (SRA) as #SRP006656. Sanger trace files from Huntington, Union Pacific, and IK-99-70 are deposited in the NCBI Trace Archive as #TI2306523713-2306523816. Consensus mitochondrial sequences are deposited in GenBank as #JF912199 (Huntington) and #JF912200 (IK-99-70). Our assemblies of Huntington, IK-99-70, and Union Pacific reads and traces are available at [61].

### Phylogenetic analyses

Detailed description of phylogenetic analyses performed can also be found in Additional file 1. These explored topological and chronological features of mammoth mitochondrial phylogeny using a Bayesian approach, comparing hundreds of sequences from a number of studies discussed above as well as from [62]. We employed jModelTest v.0.1.1 [63] to choose model parameters and BEAST v.1.5.6 [64] to build trees and estimate coalescent dates, using tip calibration points corresponding to radiocarbon ages of the samples, as well as root calibration points described by [65]. These runs were analyzed in Tracer v.1.3 [66] and trees were visualized with FigTree v.1.3 [67].

## Abbreviations

^14^Cya, Radiocarbon years ago; CM, Columbian mammoth, *Mammuthus columbi*; ILS, incomplete lineage sorting; MRCA, most recent common ancestor; mtDNA, mitochondrial DNA; PCR, polymerase chain reaction; WM, woolly mammoth, *Mammuthus primigenius*.

## Competing interests

The authors declare that they have no competing interests.

## Authors' contributions

JE, RD, DO, DF, RM and HP planned and designed the project. JE, RD, AD and CK performed wet laboratory work. JE, RD, AD, TT, and SS performed assembly and analysis of the sequence data. JE wrote the paper with assistance from all authors.

## Supplementary Material

Additional File 1 **Additional materials and methods**. A detailed description of Materials and methods. Click here for file

Additional File 2 **Additional tables**. A collection of tables referred to in the text as tables S1 through S7. Click here for file

Additional File 3 **Additional figures**. A collection of figures referred to in the text as Figures S1 to S8. Click here for file
