# Supplementary material for: Complete Columbian mammoth mitogenome suggests interbreeding with woolly mammoths
Source: Genome Biol. 2011 May 31;12(5):R51. doi: 10.1186/gb-2011-12-5-r51 (PMC3219973; doi:10.1186/gb-2011-12-5-r51)
Supplement: Additional File 1 — Additional materials and methods. A detailed description of Materials and methods. [file gb-2011-12-5-r51-S1.DOC]

**ADDITIONAL DATA FILE 1: Materials and Methods**

**Complete Columbian mammoth mitogenome suggests interbreeding with woolly mammoths**

**JACOB ENK,*^,1^ ALISON DEVAULT,^1^ REGIS DEBRUYNE,^1,2^ CHRISTINE E. KING, ^1^ TODD TREANGEN,^3^ DENNIS O’ROURKE,^4^ STEVEN L. SALZBERG,^3^ DANIEL FISHER,^5^ ROSS MACPHEE,^6^ and HENDRIK POINAR*^,1^**

^1^McMaster Ancient DNA Centre, Department of Anthropology, McMaster University, 1280 Main Street West, Hamilton, Ontario L8S 4L9, Canada

^2^Muséum national d'Histoire naturelle, UMR 7206 Eco-anthropologie, Equipe "génétique des populations humaines," 57 rue Cuvier, CP139, 75231 Paris Cedex 05

^3^Center for Bioinformatics and Computational Biology, 3115 Biomolecular Sciences Bldg #296, University of Maryland, College Park, MD 20742

^4^Department of Anthropology, University of Utah, 270 S. 1400 East Room 102, Salt Lake City, UT 84112-0060

^5^Museum of Paleontology and Department of Geological Sciences, University of Michigan, 1109 Geddes Ave. , Ann Arbor, MI 48109-1079

^6^Division of Vertebrate Zoology, American Museum of Natural History, Central Park West @ 79^th^ St, New York, NY 10024

*Corresponding authors:

Jacob Enk (enkjm@mcmaster.ca) or Hendrik Poinar (poinarh@mcmaster.ca)

McMaster Ancient DNA Centre, CNH 524

McMaster University

1280 Main St. West

Hamilton, Ontario L8S 4L9, Canada

P: 1+ 905.525.9140 x26331; F: 1+ 905.522.5993

Keywords: ancient DNA; mammoths; phylogenetics; Pleistocene; North America

**All literature cited refer to those in the main manuscript.**

**1. Laboratories**

**McMaster Ancient DNA Centre (“MAC;” McMaster University, Hamilton, Ontario, Canada)** [45]**:** Sample extraction, qPCR and PCR reactions, cloning, and Sanger sequencing reactions on Huntington, Union Pacific, and MPC IK-99-70; library preparation for and sequencing on the 454 GSFLX (454 Life Sciences, Brantford, CT, USA) of MPC IK-99-70; sequence assembly and analyses.

**Institute for Molecular Biology and Biotechnology Laboratory (“Mobix;” Hamilton, Ontario, Canada)** [47]**:** Sanger sequencing on ABI-3730 DNA Analyzer (Applied Biosystems) of sequencing reactions generated at MAC.

**Service de Systématique Moléculaire of the Muséum national d'Histoire naturelle (“MNHN;” Paris, France)** [46]**:** Replication experiments on Huntington, including sample extraction, PCR reactions, cloning, and Sanger sequencing on ABI-37 DNA analyzer (Applied Biosystems Inc., Foster City, CA, USA).

**Ambry Genetics (“Ambry;” Aliso Viejo, California, USA)** [48]**:** Illumina library preparation and evaluation, library qPCR, high throughput sequencing on the Illumina GAII platform (Illumina Inc., San Diego, CA, USA).

**Center for Bioinformatics & Computational Biology (“CBCB;” University of Maryland, College Park, Maryland, USA)** [49]**:** High-throughput sequence assembly and analyses.

**2. Operating procedures**

Pre-PCR laboratory work was performed in dedicated cleanroom facilities (MAC and MNHN), following standard protocols for attire and handling [21]. Pre- and post-PCR work were performed in physically separate laboratories. Control reactions containing no mammoth material were used in all appropriate experiments (extraction and PCRs) in order to detect contamination by previously extracted or amplified mammoth DNA. Any experiments with positive amplification in blank reactions were wholly excluded from downstream use. All PCR products were cloned before Sanger sequencing. Consensuses of Sanger sequences were derived from at least two clones from each of at least two replicate PCR products from the same extraction.

Primers used in Materials and Methods sections 4 and 5 (Additional data file 2: table S1) have been published previously [11] or were newly designed using the Integrated DNA Technologies (IDT) SciTools OligoAnalyzer 3.1 [50]. These were provided by IDT (Coralville, IA, USA; 25nmol, standard desalting) and tested for sensitivity and PCR conditions in their working pairs using copy number standards of a straight or cloned *M. primigenius* PCR products of known sequence (hapolotype D1). Primers used in section 6 (Additional data file 2: table S5) have been published previously [20] or were newly designed, ordered from IDT, and optimized in section 6b.

“*Taq*” and “PCR Buffer” hereafter refers to reagents supplied with the Ampli*Taq* Gold® (Applied Biosystems Inc., Foster City, CA, USA) DNA polymerase.

“SYBR” refers to SYBR Green® (Invitrogen, Carlsbad, CA, USA).

“BSA” refers to bovine serum albumin.

“Water” used in laboratory experiments refers to UV-sterilized, purified water.

For all mitogenomic base positions, we use the sequence obtained by Krause et al. [22] as a reference.

Sequence alignments are available at [61]

**3. Sample Selection**

Please see the main text for description of specimens.

**4. Huntington Mammoth Whole Mitochondrial Genome**

**4a. DNA Extraction**

Roughly 0.98g of tusk material from Huntington was sequestered and crushed to fine particles with a hammer. These were demineralized and digested in separate steps using buffers and incubation temperatures/durations described elsewhere [11]. This was repeated in four separate rounds of demineralization+digestion (5+5mL, 5+5mL, 3+3mL, 2+3mL) on the same tusk material, though we only used solutions from the final round for all subsequent work. Demineralization and digestion supernatants from this last round were pooled and then purified using phenol:chloroform:isoamyl alcohol (25:24:1), and resultant aqueous phases were purified again with chloroform. The final aqueous phase was concentrated by ultrafiltration with Amicon Ultra 30K columns (Millipore) and eluted in 150µL 0.1X TE (pH 8.0). We hereafter refer to the purified extract from this last round of demineralization/digestion as “*HUNT1*.”

**4b. Extraction qPCR Screen**

We used a quantitative PCR of a 79bp mammoth-specific amplicon (using primers #3+4, Additional data file 2: table S1) to estimate the amount of target mammoth DNA in *HUNT1* and screen for contamination in its associated extraction blank.

Each 20µL qPCR included: 1X PCR buffer, 2.5 mM MgCl_2_, 1mg/mL BSA, 250 µM each dNTP, 200 nM each primer, 2.5 units of *Taq* polymerase, 0.167X SYBR, 3µL of template DNA extract (straight and 0.1X dilutions), water for PCR blanks, or 0.1X TE used for sample dilution. Five mammoth DNA standards of known concentration (1 to 1,000 copies/µl) were included for each amplicon. Cycling conditions were: initial denaturation (95°C, 5m); 55 cycles of denaturation (95°C, 30s), annealing (62°C, 30s), and extension (72°C, 40s). Amplifications were executed and analyzed using the BioRad CFX96® (Biorad, Hercules, CA, USA) real-time PCR platform and associated software.

No mammoth DNA contamination was detected in the extraction blank. Estimated starting copy numbers of the 79bp fragment from *HUNT1* were ~213 (estimation from 1X concentration) and 238 (0.1X projection) copies per original microliter. Following the formula decribed by [24], this indicates only about 10% PCR inhibition, significantly lower than the same measures derived from another extraction from the same substrate (*HUNT2*, section 5e). Since *HUNT1* derives only from the last round of demineralization and digestion applied to the sample, and *HUNT2* from the first (and only) round from a different subsample of the same substrate, we expect that many of the inhibitory constituents associated with this specimen were removed in the first three rounds of the extraction described in section 4a. While this is purely hypothetical, such a strategy (pre-demineralization/digestion) may therefore prove useful for highly inhibited samples of various kinds.

**4c. DNA Size Distribution Measurement**

We performed a qPCR-based evaluation amplifiable length distribution, and thus DNA fragmentation, in *HUNT1* following the procedure designed by [52] and [24]. We amplified three incrementally longer amplicons within the woolly mammoth mitochondrial 12S gene, using a single forward primer (#15) and three different reverse primers (#16-18) (Additional data file 2: table S1), in individual singleplex reactions.

Each 20µL qPCR included: 1X PCR buffer, 2.5 mM MgCl_2_, 1mg/mL BSA, 250 µM each dNTP, 200 nM each primer, 2 units of *Taq*, 0.167X SYBR, 3µL of template DNA extract (0.1X dilution) or water for PCR blanks or 0.1X TE used for sample dilution. Four mammoth DNA standards of known concentration (1 to 1,000 copies/µl) were used for each amplicon, in replicate. Cycling conditions were: initial denaturation (95°C, 5m); 50 cycles of denaturation (95°C, 25s), annealing (62°C, 25s), and extension (72°C, 25s). The extract was amplified in triplicate. Amplifications were executed and analyzed using the BioRad CFX96® real-time PCR platform and associated software.

Estimated starting molecule counts per original microliter (averaged among triplicates) are reported in Additional data file 2 (table S2). These were log transformed and plotted against fragment length. From this, a regression line was calculated, the slope of which (λ) correlates to the rate of DNA fragmentation in the extract, and the inverse of which estimates the average amplifiable fragment size in the sample, and finally the x-intercept of which (*y=0*) estimates the maximum amplifiable fragment size. Additional data file 2 (table S2) reports the results of this evaluation, with the results from similar evaluations of other extracts used in this project and of woolly mammoth remains analyzed by for comparison. A graphical presentation of log-transformed plots is shown in Additional data file 3 (figure S2).

These results, discussed further in Section 5e, guided size selection during library preparation.

**4d. Library Preparation**

We sent *HUNT1* to Ambry Genetics for library preparation for the Illumina (Illumina Inc., San Diego, CA) platform, for which they followed the standard protocol with some proprietary modifications. Because our qPCR evaluation of the extract suggested a maximum amplifiable fragment length of only ~150bp (see above), Ambry selected only the size fraction that included 50-125bp original fragment length for sequencing. While selecting a narrower size fraction (e.g., 50-70bp) may have at least theoretically maximized the ratio of target:nontarget DNA, we expanded our selection window to balance our target sequencing goal with a desire to explore the metagenomic content of the sample for taphonomic purposes, as well as the capacity of the sequencing platform. Ambry then finalized the library enrichment by amplifying the mentioned size fraction, which provided a final total DNA content of roughly 9.28ng/ul in the library, as measured with an Agilent 2100 Bioanalyzer (Agilent).

**4e. Library qPCR Evaluation**

In order to estimate the amount of target DNA in the library prepared by Ambry, they performed a quantitative PCR experiment using an identical protocol used in the size distribution measurement, targeting just the smallest amplicon in that set (primers #15+16, table 1, Section 4c). This provided an estimate of about 3750 copies per microliter of the 63bp target in the library. Assuming that the qPCR measured only template molecules that were fully adapted, this should correspond to a molecular weight of the target amplicon of at least 5.95E-7ng/μL. By dividing the length of a whole mitochondrial genome (~16,800bp) into 63bp fragments (=~267), we conservatively estimated that ~800k similar fragments, totaling 1.55E-4ng/μL, should comprise target DNA. Since this total molecular weight is roughly 1.71E-3% of the total DNA concentration, then we predicted that at least that proportion of the eventually sequencing reads would be target. If we acquired roughly 20m reads in the sequencing run, this projects that we would obtain about 342 total 63bp reads, which would amount to roughly 1.3X *duplicate* coverage of the whole mitochondrial genome. While this model and projection clearly does not account for the shorter average read length projected from the size distribution measurement (~35bp, section 4c), we predicted that it would only underestimate the eventual read coverage. Therefore we opted to sequence the library without any further enrichment.

**4f. Sequencing with Illumina**

The aforementioned library size fraction was sequenced using the 54bp singleton protocol on the Illumina GAII platform (Illumina Inc., San Diego, CA, USA), following standard procedures with slight modification by Ambry.

**4g. Data Processing & Statistics**

Ambry performed preliminary data processing, including quality filtering and base calling. A total of ~28.5m final reads were generated from the experiment. Final FASTQ sequence read data files were sent to MAC and CBCB for subsequent assembly and analysis.

**4h. Sequence Assemblies**

Three software programs were used in sequence assembly, each using slightly different protocols.

FASTQ read files were first converted to FASTA format and then assembled to the mammoth reference genome [20] using the 454 GS Reference Mapper software (454 Life Sciences, Brantford, CT, USA) under the default parameters. A total of 7784 (7614 unique) reads successfully aligned, resulting in a 22.5X average read depth per base, with at least 2X unique read depth for all bases except the seven most 5’ bases and the nine most 3’ bases of the reference, as well as some sections of the VNTR. This corresponds to ~1.02X duplicate read depth per base, lower than expected 1.3X (section 4e). Consensus contigs were generated following assembly using the default parameters.

We also assembled the mitochondrial reads with AMOScmp [53], a program designed for comparative sequence assembly. The AMOScmp-shortReads pipeline was used, specifically designed to handle cases with short (< 100 bp) reads. AMOScmp first aligns the reads to a reference sequence with NUCmer [54], a widely-used alignment program for efficient pairwise DNA alignment. AMOScmp-shortReads parameters were configured as follows: MINCLUSTER = 16, MINMATCH = 16, MINLEN = 31, --MAXMATCH, MINOVL = 10, MAXTRIM = 18, MAJORITY = 50, CONSERR = 0.06, ALIGNWIGGLE = 2. These values resulted in 8048 reads mapping successfully to the reference mammoth genome. The average depth of coverage (in unique reads) of this assembly was 23X. The consensus was generated using the AMOS make-consensus program. In attempt to increase sensitivity, we also used the AMOScmp-shortReads-alignmentTrimmed pipeline, which increased the total mapped reads to 10962 and read coverage to 30X. The final assembly was manually inspected using amosvalidate [56] and the graphical assembly viewer Hawkeye [57]; visual inspection revealed 39 identical clones assembled to positions 1601 – 1628, which were identical to the reference in the first 20 bases but included an insertion and C>T transition in the latter portion (GTTGGCTTGGAAGCAGCCATTCATTTAA). Upon a BLAST search of this sequence, it was found to be a 100% match to several bacterial entries. This combined with the deep clonal depth lend evidence the sequence derives from non-endogenous DNA, and thus we manually removed them from the assembly. The final assembly contained two contigs gapped by the VNTR region (positions 16157 – 16476). Gaps in alignment were closed by evenly distributing aligned reads across the region, in agreement with the average depth of coverage for the assembly.

Reads from the FASTQ files were also assembled in Geneious 5.1.7 [55] to the mammoth reference and to an Asian elephant mitochondrial genome [20] using both the “low” and “medium” sensitivity levels, with no alignment fine tuning. The resultant to-elephant assemblies included several sections with no read coverage, which is consistent with the significant divergence between that genus and *Mammuthus* as assayed for woolly mammoths. From these assemblies we generated consensuses using the strict 50% threshold, with the highest quality score from all reads aligned to each base used to determine base quality, and with “N” assigned to those bases with quality scores less than 20.

A comparison of all assembly consensuses (Additional data file 2: table S3) reveals broad agreement between the to-mammoth assemblies and the medium sensitivity to-elephant assembly consensuses for those bases where they overlap. However, this to-elephant assembly consensus yields a number of disagreements with the other consensuses in a short region (positions 13707-13750), derived from a single read for that section (not included in Additional data file 2 (table S3). While these base calls are thus unlikely to be accurate, they technically bring the identity between this consensus and the to-mammoth consensuses to 99.98% excluding the VNTR. The low sensitivity to-elephant assembly, on the other hand, revealed a number of disagreements with the other assemblies, again largely corresponding to base positions with low coverage.

Broad agreement among consensuses generated from various software packages and parameters lead us to use the consensus generated from the AMOScmp assembly (Supplementary Materials: Alignment 1) as the final sequence for the Huntington mammoth. However, owing to the short read lengths, we consider the VNTR (positions 16157 – 16476) unresolvable with this data, and thus remove it from our reported consensuses, following Gilbert *et al*. [10].

**5. Sanger Sequencing of Huntington and Union Pacific mtDNA**

**5a. DNA Extraction**

We subsampled 100mg of bone (Huntington) and tooth (Union Pacific) material using bleach- and heat-sterilized tools (chisel and/or rotary tool) and then crushed them to fine particles/powder with a hammer. Samples were extracted according to procedures described elsewhere [11], except that demineralization and digestion supernatants were combined prior to the PCI purification stage. Aqueous phases of PCI processing were concentrated and reconstituted in 50µL 0.1X TE (pH 8.0) by ultrafiltration with Microcon YM-30 columns (Millipore, USA). Extractions included blanks to detect contamination. These we refer to as *“HUNB1*” for the Huntington bone and “*UPT1*” for the Union Pacific molar tooth extractions.

A second round of extractions used 1g of tusk from Huntington (“*HUNT2*”) and 0.68g of tooth root from Union Pacific (“*UPT2*”). We used these extracts for a preservation evaluation as well as to provide a single amplicon for sequencing (see below). This extraction protocol followed the same procedure referenced above, except for relative volume increases of EDTA (10ml), and digestion buffer (7–8ml). Final aqueous phases were concentrated to a final elution volume of 100µL.

**5b. PCR Amplification**

*HUNB1*, *HUNT2*, and *UPT1* were used for PCR amplification of specific targets for cloning and Sanger sequencing.

Each 20µL PCR included: 1X PCR buffer, 2.5 mM MgCl_2_, 1mg/mL BSA, 250 µM each dNTP, 200 nM each primer, 5 units of *Taq*, 0.167X SYBR, 3-5µL of template DNA extract (1X or 0.1X dilutions) or water for PCR blanks. At least one PCR blank was included per PCR reaction. Cycling conditions were: initial denaturation (95°C, 4-7m); 45-60 cycles of denaturation (95°C, 30s), annealing (59.5-62.5°C, 30s), and extension (72°C, 40s); and a final extension (72°C, 10m). Each extract was amplified in duplicate. Amplifications were executed and analyzed using the Stratagene Mx3000P® real-time PCR platform.

Amplification products were run on 2% (w/v in 0.5X TBE) agarose gels containing 1% ethidium bromide at a concentration of 1.5µL/50mL and visualized with UV light. No contamination was observed in any reaction blanks. When primer dimers and secondary products were apparent, 10 µL were rerun on 2% gels and the target amplicon was excised and dissolved in 50µL 1X TE. These were re-amplified using 2µL of the dissolved gel solution under the same conditions as the original PCR, with 1 unit of *Taq*. See Additional data file 2 (table S4) for a summary of amplification conditions and results.

**5c. Cloning and Sequencing**

All PCR products were cloned using the TOPO-TA cloning kit with “One Shot” TOP-10 chemically-competent cells (Invitrogen), using one-quarter scale reactions. Prior to cloning, older PCR products were re-adenylated in reactions containing 250µm dATP mix, 2.5mM MgCl_2_, 1X PCR Buffer, 1 unit *Taq* , water, and 3µL of PCR product, at 94°C for 7 minutes and 72°C for 20 minutes.

Colonies were chosen using blue/white selection from culture plates (containing 50mg/L ampicillin and 20µg/mL X-Gal), lysed in 50µL of 10mM Tris-Cl at 95°C for 5 minutes. 30µL colony PCR reactions were performed containing 2µL of colony lysate supernatant with “M13” primers [following [11] with a 57°C annealing temperature], and purified over 96-well 30K Acroprep filter plates (Pall Corporation, Port Washington, NY, USA) into 0.1X TE. 7µL sequencing reactions were performed following [11] using 0.3µL BigDye terminator v1.1 (Applied Biosystems), 1µL of diluted purified colony PCR product, 0.1X TE, water and the M13 forward primer. Cycled reactions were sequenced by Mobix.

Cloned sequences were manually aligned in BioEdit v7.0.9, and consensus at each position was determined by at least two clones from at least two independent PCRs. We sequenced additional clones from the same and/or additional PCRs until consensus was achieved. Sequence alignments can be found in Supplementary Materials: Alignment 4.

**5d. Sequence Replication**

In order to independently verify the sequences obtained at MAC, two subsamples from the Huntington mammoth (one from bone the other from tusk) were sent to MNHN, where they were analyzed in the “ancientDNA Box”. Roughly 100mg of each sample were processed using the same extraction procedure described in section 5a. Only a few steps diverge from the extraction protocol used at MAC: 10% sarcosyl was replaced with 2% pre-warmed SDS, and Acroprep 30K columns (Pall, USA) were used for final concentration of the extracts in 100µL of 1X TE.

Two successful amplifications performed at MAC were attempted at MNHN: primers #3+4 and #9+12 (Additional data file 2: table S1). Straight amplifications from the extracts remained unsuccessful after 3 trials. However, after the implementation of a multiplex 15-cycle pre-amplification using a combination of the four primers, secondary PCRs using 2 µL of straight pre-amplified products yielded positive products for each amplicon twice independently. PCR conditions were identical to those used at MAC except that they were performed using the SsoFast supermix (Biorad, Hercules, CA, USA) and with only 0.4mg/mL BSA. Amplification products were blunt-ended and individually cloned using the TransformAid and CloneJET PCR cloning kits (Fermentas, Estonia). Positive colony PCR products were subsequently sequenced on the ABI-37 DNA analyzer (Applied Biosystems). Four separate clones, from 2 independent PCR for each of the two fragments were sequenced and yielded the same consensus sequence as the one obtained at MAC for both amplicons.

Sequencing traces are available at [61].

**5e. Preservation Evaluation**

*HUNT2* and *UPT2* were evaluated for DNA preservation in a similar fashion as was done on *HUNT1* (section 4c). However, these qPCRs used a 63bp target (primers #6+8), an 85bp target (#5+7), and a 121bp target (#5+8) (Additional data file 2: table S1).

Each 20µL PCR included: 1X PCR buffer, 2.5 mM MgCl_2_, 1mg/mL BSA, 250 µM each dNTP, 200 nM each primer, 1-2 units of *Taq*, 0.167X SYBR, 3µL of template DNA extract (1X, 0.1X, and 0.02X dilutions) or water for PCR blanks. Five mammoth DNA standards of known concentration (1 to 10,000 copies/µl) were added as standards for each amplicon. Cycling conditions were: initial denaturation (95°C, 7m); 45 cycles of denaturation (95°C, 30s), annealing (61°C, 30s), and extension (72°C, 40s); and a final extension (72°C, 10m). Amplification properties were evaluated using the analysis software provided with the Stratagene Mx3000P® real-time PCR platform.

When combined with similar analyses performed on the extracts used in the Illumina sequencing (section 4c, Additional data file 2: table S2, Additional data file 3: figure S2), these data demonstrate that the Columbian mammoth extracts, as expected for remains from temperate contexts, exhibit significantly more highly fragmented DNA compared to permafrost-preserved woolly mammoth extracts.

**6. Whole Mitogenome of IK-99-70**

To obtain the whole mitogenomic sequence from this specimen, we employed a technique similar to Krause *et al.* [20], using three groups of primer pairs in multiplex amplification, followed by amplification from the multiplexes using each pair individually (“singleplex”), followed by purification, pooling, and sequencing on the Roche 454 GS-FLX (454 Life Sciences, Brantford, CT, U.S.A). To fill gaps in the 454 data, we used multiplexes, singleplexes, and/or original extract to reamplify necessary amplicons, which we then cloned and Sanger sequenced using similar procedures as in Section 5c.

**6a. DNA Extraction**

We used the same extract generated by [11] at 0.5X or 0.25X concentration (diluted in 0.1X TE) for all multiplex and fill-in reactions contributing to the final sequence for the specimen.

**6b. Multiplex Primer Design & Optimization**

Most primers used for this section of the project were taken directly from Krause *et al.* [20], while others we designed using the Integrated DNA Technologies (IDT) SciTools OligoAnalyzer 3.1 [50], as reported in Additional data file 2 (table S5). These comprised 47 overlapping pairs, 46 of which were initially divided into two groups (A and B) such that no pairs in the same group would amplify overlapping fragments. Later, group A was divided into two separate groups (A-1 and A-2) based on optimal annealing temperature, discussed below.

In order to determine optimal annealing temperatures for the multiplex reactions, we performed a series of PCRs (including a fluorescent dye but without DNA standard) on an exceptional mammoth extract [51] using each multiplex primer pool.

Each 20µL PCR included: 1X PCR buffer, 2.5 mM MgCl_2_, 1mg/mL BSA, 250µM each dNTP, 150nM each multiplex primer pool (each primer equilibrated), 2.5 units of *Taq*, 0.167X SYBR, 4µL of template DNA or water for PCR blanks. Cycling conditions were: initial denaturation (94°C, 9m); 30 cycles of denaturation (94°C, 30s), annealing (52-65°C, 30s), and extension (72°C, 30s); and a final extension (72°C, 10m), concluding with a melt curve from 68-95°C. Amplification properties were evaluated using the analysis software provided with the Stratagene Mx3000P® real-time PCR platform.

PCR products were also run on ethidium bromide-stained 2% agarose gels. Three properties contributed to determination of optimal annealing temperature: amplification Cq as determined by the analysis software, peak fluorescence, and visual quality/intensity of the products on agarose gels. Multiplex group A did not amplify as well as group B, therefore we split group A into two subgroups (A-1 and A-2, Additional data file 2: table S5) based on theoretical expected annealing temperatures, and determined the optimal annealing temperatures for these subgroups using identical procedures as outlined above.

We performed similar optimization experiments for each individual primer pair, amplifying from dilutions of the associated multiplexes generated beforehand.

Each 20µL PCR included: 1X PCR buffer, 2.5 mM MgCl_2_, 1mg/mL BSA, 250µM each dNTP, 1.5µM each primer, 0.5 units of *Taq*, 0.167X SYBR, and 2µL of a 0.025x dilution (in water) of the appropriate multiplex pool (optimal temperature-amplified reaction only) or Water for PCR blanks. Cycling conditions were: initial denaturation (94°C, 9m); 35 cycles of denaturation (94°C, 30s), annealing (54-65°C gradient, 30s), and extension (72°C, 40s); concluding with a melt curve from 55-95°C. Amplifications were executed and analyzed using the BioRad CFX96® real-time PCR platform and associated software.

Amplification properties were evaluated using identical metrics as described above for the multiplexes, which allowed us to determine appropriate annealing temperatures for each primer pair, indicated in Additional data file 2 (table S5). Primer pair A8a is the only exception, as it was designed for amplification solely from extract and its optimal annealing temperature was not determined. Rather we amplified this pair using the indicated (Additional data file 2: table S5) annealing temperature, which was slightly above its calculated melting temperature.

**6c. Multiplex Amplification**

All multiplex amplifications were performed in duplicate, using the optimal annealing temperatures determined from the experiments outlined above.

Each 20µL PCR included: 1X PCR buffer, 2.5 mM MgCl_2_, 1mg/mL BSA, 250µM each dNTP, 150nM each multiplex primer pool with each individual primer at 1uM, 2.5 units of *Taq*, 0.167X SYBR, 3.5-4µL of template DNA extract (at 0.5X concentration) or water for PCR blanks. Cycling conditions were: initial denaturation (94°C, 9m); 28-30 cycles of denaturation (94°C, 30s), annealing [57°C, 60°C or 58.5°C (multiplex groups A-1, A-2, or B, respectively), 30s]; and extension (72°C, 40s), concluding with a melt curve from 65-93°C. Amplifications were executed and analyzed using the BioRad CFX96® real-time PCR platform and associated software.

Successful reactions were subsequently diluted to 0.04X concentration (in water) and used as template for subsequent singleplex reactions.

**6d. Singleplex amplifications**

Using each replicate of each multiplex as template (at 0.04X concentration, diluted in water), we targeted amplicons using each primer pair in individual amplifications.

Each 20µL PCR included: 1X PCR buffer, 2.5 mM MgCl_2_, 1mg/mL BSA, 250 µM each dNTP, 1500 nM each primer pair, 0.5 units of *Taq*, 0.167X SYBR, 2µL of a 0.04X dilution (in Water) of the appropriate multiplex, or Water for PCR blanks. Cycling conditions were: initial denaturation (94°C, 9m); 29-35 cycles of denaturation (94°C, 30s), annealing (each appropriate temperature (Additional data file 2: table S5), 30s), and extension (72°C, 40s); concluding with a melt curve from 68-91°C. Amplifications were executed and analyzed using the BioRad CFX96® real-time PCR platform and associated software.

Successful PCRs were subsequently purified over 96-well 30K Acroprep filter plates (Pall Corporation, Port Washington, NY, USA) and eluted in 40µL 0.1X TE. Since one amplicon (A3) showed two distinct melt peaks and gel bands, we ran the entire product on 2% agarose gel, plugged and kept the larger (target-sized) band, which we then purified using the QIAquick™ Gel Purification Kit (QIAGEN) eluting in 50µL EB.

In total this provided 92 total purified amplicons, 46 in duplicate from independent multiplex reactions. We attempted to quantify each purified amplicon using the quantitative plate read function of the Mx3000P® real-time PCR platform on a solution of each purified amplicon and 0.167X SYBR, with a standard of known DNA quantity (Quant-iT™ PicoGreen®, Molecular Probes Inc.). We used these quantitations to pool the products in ostensibly equimolar concentrations. However, from the sequence data, it became clear that the quantitation procedure required further optimization (see below).

**6e. Library Preparation**

Each replicate amplicon pool was concentrated using Microcon YM-30 columns (Millipore, USA) to 50µL in 10mM Tris-HCl. We used 26µL of this with the Roche/454 GS FLX Titanium shotgun library preparation kit (454 Life Sciences, Brantford, CT, USA), replacing the standard A-adaptor with those having a 10bp multiplex identifier tag. We subsequently quantified the number of adapted molecules using a qPCR procedure described elsewhere [51], which allowed us to determine the necessary amount to use in emulsion PCR. Since these samples accompanied others on the same sequencing lanes, we only added enough, by our estimates, necessary for 20X clonal coverage of each amplicon.

**6f. Sequencing on 454 GS FLX & Data Processing**

Pooled libraries underwent emulsion PCR, and emulsion beads bearing successful amplification were isolated for sequencing. These were packed onto a 2-region PicoTitrePlate™ and sequenced on the 454 GS-FLX sequencer. Sequencing image files were processed using the standard shotgun image processing algorithm included in the GS software package (v2.1). This provided two individual SFF files containing those reads that passed the default quality filters.

**6g. Sequence Assembly & Fill-In**

SFF files from the two regions, which each contained a replicate amplicon pool, were sorted by their appropriate MID (allowing 2 errors) using the “sfffile” command structure in the 454 GS software package (v2.3). This produced two SFF files with 4001 and 2345 sequences for replicates 1 and 2, respectively. We then used the GS Reference Mapper (454 Life Sciences, Brantford, CT) to assemble these reads to a woolly mammoth mitochondrial genome sequence [20]. An evaluation of the results of the mapping indicated very wide variation in coverage, ranging from 0 to ~350X, probably owing to poor equilibration among amplicons in the pools used for the library generation. Regions corresponding to amplicons A3 and B19 (Additional data file 2: table s5) had no or very low (<3X) read coverage, and so we targeted these for PCR amplification and Sanger sequencing to achieve sufficient coverage for consensus determination.

Primer pair B19 was reamplified from the original group B multiplex reaction (replicate 1) for the specimen, using identical procedures as for the original singleplexes. We reamplified primer pair A3 from the original purified post-multiplex singleplex reaction, for which we used the following PCR protocol:

Each 20µL PCR included: 1X PCR buffer, 2.5 mM MgCl_2_, 1mg/mL BSA, 250µM each dNTP, 1.5µM each primer, 0.5 units of *Taq*, 0.167X SYBR Green, 5 µL of the purified original singleplex reaction or water for PCR blanks. Cycling conditions were: initial denaturation (94°C, 9m); 24 cycles of denaturation (94°C, 30s), annealing (each appropriate temperature (Additional data file 2: table S5), 30s), and extension (72°C, 40s); concluding with a melt curve from 68-91°C. Amplifications were executed and analyzed using the BioRad CFX96® real-time PCR platform and associated software.

Successful products were then cloned and Sanger sequenced as described in section 5c.

Sanger sequence chromatograms from the fill-in reactions, 454 reads in the MID-sorted SFF files, and all multiplex primers were viewed and reassembled to the mammoth mitogenome reference using Geneious Pro® v5.1.7 [55]. The assembly parameters included a custom sensitivity level (Allowing gaps of maximum 40%; maximum gap size 100bp; word length 8; index word length 8; maximum mismatches 40%; maximum ambiguity 16) with maximum fine tuning of alignment. Reads were then vertically sorted by position, and each read was then vertically sorted manually such that reads derived from the same amplicon were aligned together. We then manually trimmed the primer sequences from each read. These we then reassembled using the highest sensitivity level and maximum fine tuning, to arrive at final assemblies.

From these trimmed assemblies we built 50% consensus sequences for each replicate and compared them. At some positions, the base calls disagreed between replicates, often in regions with low (<10X) coverage. This prompted a second round of reamplification, cloning, and Sanger sequencing to provide either more clones from each original MPX replicate (A23 and B5) which followed procedures in section 6d, or a third replicate straight from extract (B1 and B23) using the following PCR protocol:

Each 20µL PCR included: 1X PCR buffer, 2.5mM MgCl_2_, 1mg/mL BSA, 250µM each dNTP, 200nM each primer, 2.5 units of *Taq*, 3µL of template DNA extract (at 0.25x dilution in 0.1X TE) or water for PCR blanks or 0.1X TE used to dilute template. Cycling conditions were: initial denaturation (95°C, 9m); 55 cycles of denaturation (95°C, 30s), annealing (temperature indicated in Additional data file 2 [table S5], 30s), and extension (72°C, 45s).

Upon cloning and sequencing, these data combined to provide enough coverage such that each base position in the final assembly agreed among consensuses from at least two original replicates (whether originally multiplex reactions or straight extract singleplexes), as represented by at least three clones, whether 454 reads or Sanger sequences of amplified product. Upon reassembly of all appropriate clones, both replicates yielded identical consensuses, except for 11 disagreements within the VNTR, which are not included in phylogenetic analysis. The sequence from these experiments yield a consensus 100% identical to the shorter sequence obtained for this specimen in another study [11]. Given this coverage depth and agreement amongst replicates, we are confident that the mitogenome sequence obtained from this specimen is genuine. Sequence alignments for both replicates and all clones of IK-99-70 can be found in Supplementary Materials: Alignment 1 and 2.

**7. Phylogenetic Analysis**

**7a. Cytochrome *b*, tRNAs, HVR**

The first step of phylogenetic analysis was restricted to a short region of the mitogenome (positions 15006–15748) that includes the latter portion of the cytochrome *b*, tRNA-Thr and tRNA-Pro genes, and the first portion of the D-Loop. At present 126 woolly mammoths have been sequenced for this entire 743bp region, and another 80 woolly mammoths have been sequenced for the 3’ 705bp in other studies [9, 10, 12, 13, 20, 37, 51]. Sequences from these mammoths were obtained from GenBank and aligned in Geneious Pro® v5.1.3 [55] with the same section of the mitogenome sequence from Huntington, as well as with the entire 743bp from three extant elephants, representing *Loxodonta cyclotis* [37], *Loxodonta africana* and *Elephas maximus* [62].

Among all mammoths, Huntington exhibits no unique polymorphisms in this region, though it does possess a unique combination of SNPs, and thus a unique haplotype. It is assignable to clade I [9, 11] based on two definitive substitutions: a T (clade II & elephants) > C (clade I) transition at position 15044, and an A (clade II & elephants) > T (clade I) transversion at position 15059. Within clade I, they are further assignable to haplogroup C, sharing polymorphisms that are present in the majority of individuals from that haplogroup (Additional data file 2: table S6). The Union Pacific Mammoth yields an identical sequence where it overlaps with the Huntington sequence (positions 15006–15062, 15119–15156, and 15611–15748).

We used the software package BEAST v. 1.5.6 [64] to discern the phylogenetic position of Huntington among other mammoths based on the 743bp region. These analyses were restricted to single representatives of each haplotype, which, with Huntington included, totaled 91 sequences (excluding elephants [Additional data file 2: table S7, sets 1a and 1b]) and 94 sequences (including elephants, [Additional data file 2: table S7, sets 2a and 2b]).

BEAST parameters for sets 1 & 2 (Additional data file 2: table S7) were as follows: Substitution probability matrix conformed to the Hasegawa-Kishino-Yano model, with an 8-category gamma-distributed site heterogeneity scheme and an assumed proportion of invariant sites (HKY+G8+I). This model was chosen using jModelTest v. 0.1.1 [63], in which the Bayesian Information Criteria analysis indicated its appropriate application for both datasets. Two relaxed clock models were used in separate analyses, including the uncorrelated lognormal and uncorrelated exponential models. Trees were generated using the piecewise-constant Bayesian Skyline demographic model with 20 groups. Each parameter set ran for 10m generations (sampling every 1000 generations) using the default prior distributions and operator settings, except with the “skyline.Popsize” prior set to 0 to 100. Operator analyses following these runs suggested tuning modifications, which we carried out for the subsequent runs. However, previous experience suggested that expanding the integer.RandomWalk operator to a window size of 100 (rather than the suggested 2.0) is appropriate for this dataset. Following operator adjustment, each parameter set was used in 3 independent 10m generation runs (sampling every 1000 generations). Tree files were combined using LogCombiner with a 10% sample burn-in applied to each independent run. These were then combined in TreeAnnotator using the default annotation parameters, keeping target node heights. Combined log files as viewed in Tracer v1.5 [66] yield ESS>100 for each analysis parameter, suggesting full posterior convergence for each set. Trees were viewed in FigTree v1.3.1 [67] for evaluation of topology and determination of nodal posterior probabilities.

Annotated trees from all four parameter sets are presented in Additional data file 2 (figures S3 through S6), with nodal posterior probabilities indicated. As demonstrated, nodal posterior probabilities for the clade that includes haplogroups C, D, and E (clade I) are consistently 1.00 in all analyses, indicating strong support for the inclusion of Huntington within that clade. In both variants of set 1, there is also modest support for the monophyletic grouping of all C mammoths, with posterior probabilities of 0.63 (set 1a) and 0.80 (set 1b) at the MRCA node for those haplotypes. Interestingly, in sets 2a and 2b, we observe the polyphyletic relationship between two subdivisions within haplogroup C observed in Debruyne *et al.* [11], though with only limited nodal support for monophyly of D+E and the C subgroup. While the topology within haplogroup C thus remains somewhat unresolved, we are confident that the Huntington sequence at least falls securely within clade I, and most probably within haplogroup C. Therefore we assign it an appropriate new haplotype (C32).

We also performed a set of analyses exploring temporal aspects of the tree for this mitogenomic region, using root + tip calibration and two different clock models (the uncorrelated lognormal and uncorrelated exponential models) and the Bayesian Skyline demographic model (30 groups). This was restricted to analysis of only those mammoths with finite radiocarbon dates (n = 156) and three modern elephants. For the root calibration (tMRCA for elephants + mammoths), we used 7.7my (sd=5e5), corresponding to the date calculated by Rohland et al. [65], which is also consistent with fossil evidence for the divergence of the genera. Following an initial run in order to determine appropriate operator tuning, we ran these analyses for two (exponential model) and three (lognormal model) independent 50m generation runs. The lognormal model failed to converge, yielding ESS<100 for some parameters. The exponential model did, however, converge to ESS>100 for all parameters after 100m generations. Temporal estimates on key nodes in mammoth phylogeny are reported in Additional data file 2 (table S7).

**7b. Whole Mitogenomes**

Similar analyses as above were executed using full mitogenomic sequences excluding the VNTR, which are publically available for 20 mammoths including the ones reported here. We used sequences from only one elephant of each genus as outgroups for these analyses. For both the full data set (employed in set 4) and only those with finite radiocarbon/modern dates (set 5), analyses employed the Tamura-Nei 93 substitution model with 8 gamma categories, a Bayesian Skyline demographic model (10 groups, constant addition), and tuning, burnin, combination and annotation protocols similar to section 7a.

Consensus trees and associated nodal posterior probabilities generated in the topological analyses (set 4) are depicted in Additional data file 3 (figures S7 and S8). Temporal estimates for key nodes in mammoth phylogeny are included in Additional data file 2 (table S7). As observed elsewhere [14] temporal estimates derived from analyses of full mitogenomes are significantly more ancient than the same estimates derived from analyses of shorter mitogenomic regions.

**8. Nuclear Genome Read Analysis**

**8a. Read Classification**

In order to estimate the nuclear genome divergence, we mapped all Huntington mammoth reads to *Loxodonta africana* to first determine the subset of nuclear genome reads. We used NUCmer to align reads to the reference *L. africana* assembly [60] with the following parameters: MINCLUSTER = 16, MINMATCH = 16, --MAXMATCH. This yielded 2,505,001 reads classified as nuclear, or 8.7% of the total reads. Then, to estimate divergence, we downloaded 3.6 million woolly mammoth M4 reads [59] from the SRA SRX001906 genomic fragment library. Before aligning the reads we aggressively trimmed 5 nucleotides from each end in attempt to remove divergence bias caused by sequencing errors and DNA damage. We then aligned the reads using NUCmer (same parameters) and filtered the results with show-coords to exclude any reads with <80 % ID and <35 nt in length. We also only reported unique, 1-to-1 alignments. This returned a final set of 15,650 aligned nuclear reads.

To accompany this result, we also performed individual classification of a subsample of the reads using the metagenomics analysis program PhymmBL [58] and a local sequence database including *L. africana*. Instead of running Phymm on the 28 million reads we randomly selected 1000 reads with replacement from the total sample of 28+ million reads 100 times. PhymmBL assigns a species identifier to each read, and we used this to count the percentage of reads that were classified *as L. africana*, which was 6%.

Given these two results we estimate the nuclear reads in this sample to be between 6-8% of the total.

**8b. Nuclear Genome Divergence Estimate**

In an effort to compare the Huntington and woolly mammoth nuclear genomes, we analyzed the degree of divergence between the Huntington nuclear read data and the woolly mammoth (“M4”) nuclear genome [59]. In order to limit the influence of sequencing error and mitigate effects of low coverage of the M4 mammoth nuclear genome (<<1X), we aligned only those Huntington reads to regions of the M4 nuclear genome that are covered by at least 2 reads from that dataset, which reduced the alignable Huntington reads to less than 1000. Then, after carefully inspecting the alignments returned by NUCmer using show-aligns, we determined the average percent identity of these reads to be 97% to the woolly mammoth nuclear reference. We also compared the M4 mitochondrial genome to the Huntington genome, and found it more similar (99.5% identical) to Huntington than the Krause *et al*. [20] mitochondrial genome. The differences between the nuclear and mitochondrial estimates, however, are very likely to be driven largely by the significantly lower coverage of the nuclear data (for both M4 and Huntington) and therefore more susceptible to miscalls from sequencing error and DNA damage. Thus, the real nuclear divergence between the species can only be confirmed by additional, much deeper sequencing
