# Supplementary material for: Complete Columbian mammoth mitogenome suggests interbreeding with woolly mammoths
Source: Genome Biol. 2011 May 31;12(5):R51. doi: 10.1186/gb-2011-12-5-r51 (PMC3219973; doi:10.1186/gb-2011-12-5-r51)
Supplement: Additional File 2 — Additional tables. A collection of tables referred to in the text as tables S1 through S7. [file gb-2011-12-5-r51-S2.DOC]

**ADDITIONAL DATA FILE 2: Tables S1-S7**

**Complete Columbian mammoth mitogenome suggests interbreeding with woolly mammoths**

**JACOB ENK,*^,1^ ALISON DEVAULT,^1^ REGIS DEBRUYNE,^1,2^ CHRISTINE E. KING, ^1^ TODD TREANGEN,^3^ DENNIS O’ROURKE,^4^ STEVEN L. SALZBERG,^3^ DANIEL FISHER,^5^ ROSS MACPHEE,^6^ and HENDRIK POINAR*^,1^**

^1^McMaster Ancient DNA Centre, Department of Anthropology, McMaster University, 1280 Main Street West, Hamilton, Ontario L8S 4L9, Canada

^2^Muséum national d'Histoire naturelle, UMR 7206 Eco-anthropologie, Equipe "génétique des populations humaines," 57 rue Cuvier, CP139, 75231 Paris Cedex 05

^3^Center for Bioinformatics and Computational Biology, 3115 Biomolecular Sciences Bldg #296, University of Maryland, College Park, MD 20742

^4^Department of Anthropology, University of Utah, 270 S. 1400 East Room 102, Salt Lake City, UT 84112-0060

^5^Museum of Paleontology and Department of Geological Sciences, University of Michigan, 1109 Geddes Ave. , Ann Arbor, MI 48109-1079

^6^Division of Vertebrate Zoology, American Museum of Natural History, Central Park West @ 79^th^ St, New York, NY 10024

*Corresponding authors:

Jacob Enk ([enkjm@mcmaster.ca](mailto:enkjm@mcmaster.ca)) or Hendrik Poinar ([poinarh@mcmaster.ca](mailto:poinarh@mcmaster.ca))

McMaster Ancient DNA Centre, CNH 524

McMaster University

1280 Main St. West

Hamilton, Ontario L8S 4L9, Canada

P: 1+ 905.525.9140 x26331; F: 1+ 905.522.5993

Keywords: ancient DNA; mammoths; phylogenetics; Pleistocene; North America

**All literature cited refer to those in the main manuscript.**

**TABLES**

**Table S1:** Primers and target regions used in this study. *Ref*: * = [11], ◊ = This study.

| *#* | *F/R* | *5’ position* | *5’ – 3’ sequence* | *Ref.* |  |  | | *Amplicon size (bp)* | | | | | | |
| --- | --- | --- | --- | --- | --- | --- | --- | --- | --- | --- | --- | --- | --- | --- |
|  |  |  |  |  | *63* | *78* | *79* | *85* | *86* | *103* | *110* | *118* | *121* | *124* |
| 1 | F | 14985 | GCCATCCTACGATCTGTACCA | * |  |  |  |  |  | ✓ |  |  |  |  |
| 2 | R | 15087 | GGTGTTTAGATGTATGTAGAAGTGG | ◊ |  |  |  |  |  | ✓ |  |  |  |  |
| 3 | F | 15097 | TACTTCGACCTCTTAGCCAAGT | * |  |  | ✓ |  |  |  |  |  |  |  |
| 4 | R | 15175 | GGTTGACTGCCAATTCATG | * |  |  | ✓ |  |  |  |  |  |  |  |
| 5 | F | 15337 | CTATTTTCTAAGGGTATTCAGGGAAGAG | ◊ |  |  |  | ✓ |  |  |  |  | ✓ |  |
| 6 | F | 15395 | CTGAAATTCTTCTTAAACTATTCCCTGC | ◊ | ✓ |  |  |  |  |  |  |  |  |  |
| 7 | R | 15421 | CAGGGAATAGTTTAAGAAGAATTTCAGT | ◊ |  |  |  | ✓ |  |  |  |  |  |  |
| 8 | R | 15457 | TTAATGCACGATGTACATAGCGG | ◊ | ✓ |  |  |  |  |  |  |  | ✓ |  |
| 9 | F | 15587 | CAAGTCATATTCGTGTAGATTCAC | ◊ |  |  |  |  |  |  | ✓ |  |  |  |
| 10 | F | 15647 | GATAAACCATAGTCTTACATAGCAC | ◊ |  |  |  |  |  |  |  |  |  | ✓ |
| 11 | F | 15668 | GCACATTAAAGCTCTTGATCGTACAT | ◊ |  | ✓ |  |  |  |  |  |  |  |  |
| 12 | R | 15696 | GCTATGTACGATCAAGAGCTT | ◊ |  |  |  |  |  |  | ✓ |  |  |  |
| 13 | R | 15745 | CAACCGTTGGAGGTGATATGC | ◊ |  | ✓ |  |  |  |  |  |  |  |  |
| 14 | R | 15770 | TTCTCGGAGGTAGGTAGTTAAG | * |  |  |  |  |  |  |  |  |  | ✓ |
| 15 | F | 134 | GCCAGTGAATACGCCTTCTAA | ◊ | ✓ |  |  |  |  |  |  |  |  |  |
| 16 | R | 196 | GGTGTGTGTGCTTGATGC | ◊ | ✓ |  |  |  |  |  |  |  |  |  |
| 17 | R | 219 | GCGAGACGTCATGAGCTACA | ◊ |  |  |  |  | ✓ |  |  |  |  |  |
| 18 | R | 251 | ACTACTGCTGTTTCCCGTGG | ◊ |  |  |  |  |  |  |  | ✓ |  |  |

**Table S2.** Copies/mg of substrate and resultant regression statistics, following the model proposed by [52]. *M. columbi:* includes samples of various dilutions. *(λ)* (lambda): the linear slope of the regression line calculated from the relationship between log-transformed molecular count and amplicon size. *1/λ* (inverse Lambda): estimated average amplifiable fragment length in the sample. *y=0* (x-intercept): estimated maximum amplifiable fragment size in the sample. *Since *HUNT1* comes from 5 of 31 (~16%) total mL of solution generated from the 0.98g tusk sample, we project these metrics assuming it derives from 158mg (0.16*980mg). Red = metrics derived from only 2, rather than 3 or more, data points.

|  | *Amplicon Length (bp)* | | | | | | *Λ* | *1/λ* |  |
| --- | --- | --- | --- | --- | --- | --- | --- | --- | --- |
| *M. primigenius* | *84* | *151* | *279* | *490* | *677* | *921* |  |  | *y=0* |
| Ber12 | 1,932.5 | 807.7 | 42.9 | 1.3 | 0.0 | 0.0 | -0.0080 | 125.4 | 500.3 |
| 173 | 1,536.5 | 773.9 | 69.9 | 5.1 | 1.1 | 0.3 | -0.0047 | 214.7 | 722.8 |
| 917 | 19,200.5 | 13,125.9 | 1,508.3 | 138.7 | 26.9 | 2.7 | -0.0047 | 212.3 | 985.0 |
| 473 | 864.3 | 420.5 | 54.1 | 5.1 | 0.8 | 0.0 | -0.0052 | 192.7 | 640.8 |
| 472 | 7,261.3 | 3,249.1 | 253.9 | 16.8 | 4.3 | 0.5 | -0.0050 | 200.3 | 813.0 |
| 915 | 6,095.5 | 3,870.4 | 534.1 | 57.9 | 15.7 | 1.9 | -0.0043 | 235.1 | 956.1 |
| *M. columbi* | *63* | *85* | *86* | *118* | *121* | *-* |  |  |  |
| *HUNT1* 0.1X* | 487.6 | - | 92.8 | 12.6 | - | - | -0.0287 | 34.8 | 155.7 |
| *HUNT2* 1X | 20.8 | 10.3 | - | - | 0.5 | - | -0.0289 | 34.6 | 113.0 |
| *HUNT2* 0.1X | 53.6 | 17.8 | - | - | 1.0 | - | -0.0299 | 33.4 | 123.1 |
| *HUNT2* 0.02X | 99.7 | 3.0 | - | - | 0.0 | - | -0.0692 | 14.5 | 91.9 |
| *UPT2* 1X | 2.5 | 0.9 | - | - | 0.0 | - | -0.0214 | 46.8 | 81.9 |
| *UPT2* 0.1X | 0.9 | 1.0 | - | - | 0.0 | - | - | - | - |

**Table S3.** Assembly and consensus comparisons. *Con.*: Assembly/Consensus ID. *Program*: Software program used in assembly and consensus generation. *Reference*: reference sequence for assembly. *Reads aligned*: number of reads aligning to the reference in the assembly. *Consensus Length*: length of the consensus sequence after gaps removed, including any within the VNTR. Red = disagreements from other consensuses at indicated base positions, not including gaps and anomalous sections in the *Elephas* assemblies as discussed in section 4h.

| *Con.* | *Program* | *Reference* | *Sens.* | *Reads*  *Aligned* | *Consensus Length* | 2882 | 3541 | 4774 | 4776 | 5665 | 7298 | 9710 | 10650 | 10792 | 10952 | 11514 | 11628 | 13769 | 15043 | 15257 |
| --- | --- | --- | --- | --- | --- | --- | --- | --- | --- | --- | --- | --- | --- | --- | --- | --- | --- | --- | --- | --- |
| 1 | 454 RefMap | Mammoth | - | 7784 | 16738 | C | T | T | T | C | C | T | C | T | C | C | T | C | C | G |
| 2 | AMOScmp | Mammoth | - | 8048 | 16770 | C | T | T | T | C | C | T | C | C | C | C | T | C | C | G |
| 3 | Geneious | Mammoth | Low | 6664 | 16689 | C | T | T | T | C | C | T | C | C | C | C | T | C | C | G |
| 4 | Geneious | Mammoth | Med | 7484 | 16689 | C | T | T | T | C | Y* | T | C | C | C | C | T | C | C | G |
| 5 | Geneious | *Elephas* | Low | 5137 | 16355 | Y | G | N | C | Y | C | - | T | C | T | S | N | Y | S | A |
| 6 | Geneious | *Elephas* | Med | 6682 | 16814 | C | T | T | T | C | C | T | C | C | C | C | T | C | C | G |

* Six reads cover this position; of the five unique, three call “C” while two call “T.”

**Table S4.** PCR summary. Primer pairs are indicated, referencing table S1.

| *Amplicon* | *Sample* | *PCRs.* | *Ext. vol (μl)* | *PCR product* | *Reamp?* | *Sequenced?* | *Clones Seq.* | *Init.* | *Cycles* | *T_a_ (°C)* |
| --- | --- | --- | --- | --- | --- | --- | --- | --- | --- | --- |
| 78bp  (#11+13) | *HUNT2* | 1 | 3 | + | N | Y | 3 | 4 min | 60 | 60 |
|  |  | 2 | 3 | + | N | Y | 3 |  |  |  |
| 79bp  (#3+4) | *HUNB1* | 1 | 3 | + | N | Y | 4 | 7 min | 50 | 62.5 |
|  |  | 2 | 3 | + | N | N |  |  |  |  |
|  |  | 3 | 3 | + | N | Y | 5 | 7 min | 51 | 62 |
|  | *UPT1* | 1 | 5 | + | N | Y | 2 | 7 min | 50 | 62.5 |
|  |  | 2 | 5 | + | N | Y | 3 |  |  |  |
| 103bp  (#1+2) | *HUNB1* | 1 | 3 | (+)? | N | N |  | 7 min | 50 | 62.5 |
|  |  | 2 | 3 | - |  |  |  |  |  |  |
|  |  | 3 | 3 | (+) | Y | Y | 3 | 7 min | 51 | 62 |
|  |  | 4 | 3 | (+) | Y | Y | 1 |  |  |  |
|  | *UPT1* | 1 | 5 | (+)? | N | N |  | 7 min | 50 | 62.5 |
|  |  | 2 | 5 | (+)? | N | N |  |  |  |  |
|  |  | 3 | 3 (1in10) | (+) | Y | N |  | 7 min | 51 | 62 |
|  |  | 4 | 3 (1in10) | - |  |  |  |  |  |  |
|  |  | 5 | 5 | + | N | Y | 5 | 4 min | 60 | 62.5 |
|  |  | 6 | 5 | + | N | Y | 6 |  |  |  |
| 110bp  (#9+12) | *HUNB1* | 1 | 5 | + | N | Y | 3 | 7 min | 45 | 59.5 |
|  |  | 2 | 5 | + | N | Y | 3 |  |  |  |
|  | *UPT1* | 1 | 5 | + | N | Y | 2 | 7 min | 45 | 59.5 |
|  |  | 2 | 5 | + | N | Y | 2 |  |  |  |
| 124bp  (#10+14) | *HUNB1* | 1 | 5 | (+)? | N | N |  | 7 min | 45 | 59.5 |
|  |  | 2 | 5 | - |  |  |  |  |  |  |
|  |  | 3 | 5 | - |  |  |  | 7 min | 51 | 59.5 |
|  |  | 4 | 5 | + | N | Y | 3 |  |  |  |
|  |  | 5 | 5 | (+) | Y | Y | 6 | 4 min | 60 | 59.5 |
|  |  | 6 | 5 | - |  |  |  |  |  |  |
|  |  | 7 | 5 | - |  |  |  | 4 min | 60 | 59.5 |
|  |  | 8 | 5 | - |  |  |  |  |  |  |
|  | *UPT1* | 1 | 5 | + | N | Y | 1 | 7 min | 45 | 59.5 |
|  |  | 2 | 5 | + | N | Y | 2 |  |  |  |
|  |  | 3 | 3 | + | N | Y | 3 | 7 min | 51 | 59.5 |
|  |  | 4 | 3 | + | N | N |  |  |  |  |

**Table S5.** Primers used in the multiplex PCR experiments (section 6). *Grp*: multiplex group name. *For/Rev 5’*: The 5’ position each primer. *Rf*: * = [20], ◊ = This study. *Bp*: amplicon length in nucleotides. *Ta*: annealing temperature used.

| *Grp* | *Pair* | *For 5'* | *Forward* | *Rf* | *Rev 5'* | *Reverse* | *Rf* | *Bp* | *Ta* |
| --- | --- | --- | --- | --- | --- | --- | --- | --- | --- |
| A-2 | A1 | 84 | CCGGCCTTCTTATTGGTTAC | * | 570 | GTAGTTCTCTGGCGGATAGC | * | 487 | 63.5 |
| A-2 | A2 | 1023 | TGGGTAACTCAAAGTGTAGCTT | * | 1458 | TCTGGACAACCAGCTATCATC | * | 436 | 63.2 |
| A-2 | A3 | 1876 | AGGGAAAGATTAAAAGAAGGA | * | 2361 | GGTAACTTGTTCCGTTGATCA | * | 486 | 62.0 |
| A-2 | A4 | 2585 | CTTACCAAGACGCCTTCAGC | * | 3021 | GGATATGGTATTGGAAGAGG | * | 437 | 63.2 |
| A-1 | A5 | 3371 | TTCAACGTCGAATATTCAGC | * | 3755 | CAGGGTTTAGACCTCTATAATTT | * | 385 | 61.0 |
| A-1 | A6 | 4092 | CAAGCCACAGCATCCATAAT | * | 4461 | GGGAGATTGAAGAGTAGGC | * | 370 | 61.0 |
| A-1 | A7 | 4837 | TGAAAATAACCTGACAATTTA | * | 5224 | AAGCAGCTTCAATTCTGCC | * | 388 | 56.2 |
| A-2 | A8 | 5547 | ATTATAATTGGAGGCTTTGG | * | 6021 | ATAGAATTGGGTCTCCTCCT | * | 475 | 62.0 |
| - | A8a | 5504 | CACAGCACACGCCTTTGTAA | ◊ | 6017 | AATTGGGTCTCCTCCTCCTG | ◊ | 514 | 63.5 |
| A-1 | A9 | 6397 | TTGTTCTTGCCAATTCTTCACT | * | 6855 | CTGGTTCTTCGAATGTATGA | * | 459 | 61.0 |
| A-2 | A10 | 7115 | TCCTTATTAGCTCCTTAGTCTTG | * | 7545 | AATTGCATCTGTTTTTAGACC | * | 431 | 63.5 |
| A-2 | A11 | 7724 | GCACTAACCTTTTAAGTTAGAGTAT | * | 8075 | TTTGACTAGTCATTGTTGGA | * | 352 | 57.5 |
| A-2 | A12 | 8290 | TCTCACTAGCCCATCTTCTC | * | 8670 | GATAATGCTCCGGTAAGAGGTC | * | 381 | 63.2 |
| A-1 | A13 | 9052 | CTTAATAGAAGGAAATCGTAAA | * | 9436 | CAAAGCCTACTAATTGGAAGTT | * | 385 | 56.2 |
| A-1 | A14 | 9778 | CAAAAAGGCCTTGAATGAAC | * | 10202 | GGGAATAAGTATGATTGTTGGTA | * | 425 | 61.0 |
| A-1 | A15 | 10461 | ATCCTCTTACAAGTATCCCTAAT | * | 10834 | GGTGCTTCTACATGAGCTTT | * | 374 | 63.2 |
| A-2 | A16 | 11169 | CGCATTCATAGCCGAACTAT | * | 11736 | TTTTATCTGGAGTTGCACCA | * | 568 | 63.5 |
| A-1 | A17 | 12522 | CCGCTTTTATCCATTAATAGAAA | * | 12815 | ATTTTGCGGATGTCTTGTTCG | * | 294 | 63.2 |
| A-1 | A18 | 11996 | CCCTAACATTCATGCCAATTG | * | 12421 | CAAATTGGGCTGATTTTCCT | * | 426 | 63.2 |
| A-2 | A19 | 13150 | AATACTATTCCGCATACAACAC | * | 13627 | GGTTTTGATTTTTGGCTATG | * | 478 | 61.0 |
| A-2 | A20 | 14032 | ACCACATAAAGCACACTCAT | * | 14577 | CCCCTCAGAATGATATTTGT | * | 546 | 61.0 |
| A-1 | A21 | 14802 | ATCCCCTTTCACCCGTACTA | * | 15230 | GGAGAAATATAGAATTGAGGCTA | * | 429 | 63.2 |
| A-1 | A22 | 15395 | CTGAAATTCTTCTTAAACTATTCC | * | 15736 | GAGGTGATATGCATGATGA | * | 342 | 61.0 |
| A-2 | A23 | 16086 | GCACGGTATATATGGGGTAT | * | 16534 | TGAGCCAAGGGTAACTAAGG | * | 449 | 57.5 |
| B | B1 | 522 | CCCTAAACTTTGATAGCTACC | ◊ | 1071 | CTAGGTGTAAGCCAGATGCT | * | 550 | 63.0 |
| B | B2 | 1405 | CTTACAGATAGAGGTGAAATACCA | * | 1919 | GTTTATGTTTGCCGAGTT | * | 515 | 60.3 |
| B | B3 | 2269 | AAAACCTCCGAACGATATTA | * | 2664 | AATCCTGTTCTTGGATTGG | * | 396 | 60.5 |
| B | B4 | 2973 | CCCTAACCTTAGCCCTAACT | * | 3437 | TGATAATGTTAGCGTATTCG | ◊ | 465 | 60.0 |
| B | B5 | 3683 | CCACCACAAGCATAGAAATA | * | 4157 | TCATTGTCCTGAGTATATTAGATT | * | 475 | 60.5 |
| B | B6 | 4412 | CCTAAATCAAACACAACTACGA | * | 4898 | GGTGATTAGAGTCGGTAATAT | ◊ | 487 | 58.7 |
| B | B7 | 5149 | TGGCTTCAATCTACTTCTCC | * | 5593 | CCGATTATAAGTGGAATTAATCA | * | 445 | 60.0 |
| B | B8 | 5963 | GGACCGCAACCTCAATACTAC | * | 6441 | AGGTATCATGTAGGACAATG | ◊ | 479 | 63.0 |
| B | B9 | 6762 | TCTAAGCGCGAAGTTTCT | * | 7228 | CTGGGAGAATGGTTCAGAT | * | 467 | 60.5 |
| B | B10 | 7502 | ATTCATGGGCTGTCCCAT | * | 7941 | CGTTCACTTCTTCTTTCAAGG | * | 440 | 63.0 |
| B | B11 | 8025 | AATCGCCTAATTACCAACC | * | 8330 | GAATGTAGGTGTTCCTTGTGG | ◊ | 306 | 63.0 |
| B | B12 | 8612 | CAAACACATGCCTATCACAT | * | 9102 | ATAGTAATAAGGAGGGCTTG | ◊ | 491 | 60.0 |
| B | B13 | 9297 | ACTTTGGCTTTGAAGCAG | * | 9837 | TCGAAATCATTTGTTTTGTT | * | 541 | 58.7 |
| B | B14 | 10124 | CCTACGGACTAGACTACGTACAA | * | 10541 | AAGTGTTGTTTCAAATATAATG | ◊ | 418 | 58.7 |
| B | B15 | 10767 | GCTTTCATAGTAAAAATACCTCTA | ◊ | 11230 | GCTATTAGTGGGAGAAGGGTTT | * | 464 | 63.0 |
| B | B16 | 11696 | TGGTCTTAGGCACCAAAA | * | 12051 | TGAAAATTCTATGATTGATCAGG | ◊ | 356 | 58.7 |
| B | B17 | 12323 | CCTGAGAATTTCAACAAATCTT | ◊ | 12648 | TGCGATAATCTTTTTGATGT | * | 326 | 58.7 |
| B | B18 | 12769 | GGCTCTATCATCCACAACCT | * | 13192 | GTGGAGGTATGGTTATTTGG | * | 424 | 63.0 |
| B | B19 | 13565 | CCTCAATAGCAATAAAAATACTAA | * | 14120 | CGATGGTTTTTCAGATCATT | * | 556 | 60.3 |
| B | B20 | 14527 | CCGCCTTCATAGGATATGT | * | 14926 | GCTGGTATGTAGTTGTCGGG | * | 400 | 63.0 |
| B | B21 | 15062 | ACCACTTCTACATACATCTA | * | 15543 | AATGTGATGCACGATTATACA | * | 482 | 58.7 |
| B | B22 | 15677 | AGCTCTTGATCGTACATAGC | * | 16128 | GTCCTCCGAGCATTGACT | * | 452 | 63.0 |
| B | B23 | 16478 | CCACTATGTAACTATCTCTTCAAA | * | 196 | GGAGTGTGTGCTTGATGC | * | 489 | 63.0 |
|  |  |  |  |  |  |  |  |  |  |

**Table S6.** Clade- and haplogroup-defining polymorphisms assayed for the 743bp region of the mammoth mitogenome. Base positions (sites) refer to the reference sequence [20]. Coded nucleotide bases: Uppercase = nucleotide base found in >95% of all haplotypes of the indicated group; lowercase = base found in most haplotypes (>70%) of that group; blue = experimental data from this study, with Huntington-only data indicated in standard face, and data observed in both Huntington and Union Pacific in underlined bold face. *Haplogroup B is represented by two individuals, hence the ambiguous consensus at several positions.

|  |  |  | *Clade-specific sites* | | *Haplogroup-specific sites* | | | | | | | |
| --- | --- | --- | --- | --- | --- | --- | --- | --- | --- | --- | --- | --- |
| *Clade* | *Taxon* | *Hap* | *15044* | *15059* | *15144* | *15183* | *15425* | *15568* | *15612* | *15621* | *15622* | *15625* |
| II | *M. primigenius* | *A* | T | A | t | C | G | C | G | C | T | T |
|  | *M. primigenius* | *B** | T | A | T | T | A/G | T/C | G | T/C | T/C | T |
| I | *M. primigenius* | *C* | C | T | T | T | G | T | G | T | t | T |
|  | *M. columbi* |  | **C** | **T** | **T** | T | G | T | **G** | **T** | **T** | **T** |
|  | *M. primigenius* | *D* | C | T | C | T | G | t | A | T | C | C |
|  | *M. primigenius* | *E* | C | T | C | T | A | t | A | T | C | C |

**Table S7.** BEAST analyses. *Samples:* “Elephants” = two (sets 1-3) or one (sets 4-5) *Loxodonta* sequence(s) and one *Elephas* sequence. “Mammoths” = all woolly mammoths and Huntington. “1ea hap” = one representative of each haplotype. “Dated” = remains with associated finite radiocarbon dates. *Clock:* Clock model used for each analysis, “Exp” = the uncorrelated exponential clock model. “Log” = the uncorrelated lognormal clock model; *Root Cal:* indicates whether root age prior was used, “N” = prior omitted, “Y”=normal distribution prior with mean 7.7my and standard deviation 500ky, derived from the findings of [65]. *Tip Cal:* Indicates whether or not tip dates were used. For those mammoths with associated radiocarbon dates, the raw date was used (not calendar-calibrated). For elephants, an age of 0rcya was used. Red = combined runs did not reach ESS>100 for each parameter.

|  |  |  |  |  |  |  |  |  |  |  | *tMRCA mammoths* | | | *tMRCA clade I* | | | *tMRCA haplogroup C* | | |
| --- | --- | --- | --- | --- | --- | --- | --- | --- | --- | --- | --- | --- | --- | --- | --- | --- | --- | --- | --- |
| *Set* | *#*  *Seqs* | *Bp* | *Samples* | *Clock* | *Root*  *Cal* | *Tip*  *Cal* | *Runs* | *Gens* | *Mean Posterior*  *Probability* | *Mean Tree*  *Likelihood* | *95%HPD*  *lower* | *Median*  *(years)* | *95%HPD*  *upper* | *95%HPD*  *lower* | *Median*  *(years)* | *95%HPD*  *upper* | *95%HPD*  *lower* | *Median*  *(years)* | *95%HPD*  *upper* |
| 1a | 91 | 743 | Mammoths, 1ea hap | Exp | N | N | 3 | 10m | -1814.01 | -2073.50 | - | - | - | - | - | - | - | - | - |
| 1b | 91 | 743 | Mammoths, 1ea hap | Log | N | N | 3 | 10m | -1820.96 | -2092.03 | - | - | - | - | - | - | - | - | - |
| 2a | 94 | 743 | Elephants, mammoths, 1ea hap | Exp | N | N | 3 | 10m | -2207.29 | -2434.60 | - | - | - | - | - | - | - | - | - |
| 2b | 94 | 743 | Elephants, mammoths, 1ea hap | Log | N | N | 3 | 10m | -2208.34 | -2454.13 | - | - | - | - | - | - | - | - | - |
| 3a | 159 | 743 | Elephants, mammoths, dated | Exp | Y | Y | 2 | 50m | -4848.56 | -2254.51 | 1.64e5 | **4.53e5** | 1.12e6 | 9.06e4 | **2.04e5** | 3.97e5 | 7.10e4 | **1.57e5** | 3.26e5 |
| 3b | 159 | 743 | Elephants, mammoths, dated | Log | Y | Y | 3 | 50m | -4926.59 | -2257.58 | 2.07e5 | **6.65e5** | 1.54e6 | 1.10e5 | **2.81e5** | 6.45e5 | 7.86e4 | **2.16e5** | 5.12e5 |
| 4a | 22 | 16.5k | Elephants, mammoths | Exp | N | N | 3 | 20m | -30323.57 | 30417.87 | - | - | - | - | - | - | - | - | - |
| 4b | 22 | 16.5k | Elephants, mammoths | Log | N | N | 3 | 20m | -30322.35 | 30419.93 | - | - | - | - | - | - | - | - | - |
| 5a | 18 | 16.5k | Elephants, mammoths, dated | Exp | Y | Y | 3 | 20m | -30491.91 | 30051.40 | 2.67e5 | **1.58e6** | 4.92e6 | 1.35e5 | **6.25e5** | 1.65e6 | 6.15e4 | **2.54e5** | 7.77e5 |
| 5b | 18 | 16.5k | Elephants, mammoths, dated | Log | Y | Y | 1 | 20m | -30505.31 | 30054.62 | 1.02e6 | **1.65e6** | 2.46e6 | 3.85e5 | **6.12e5** | 8.87e5 | 2.18e5 | **4.09e5** | 6.25e5 |
