# Supplementary material for: Complete Columbian mammoth mitogenome suggests interbreeding with woolly mammoths
Source: Genome Biol. 2011 May 31;12(5):R51. doi: 10.1186/gb-2011-12-5-r51 (PMC3219973; doi:10.1186/gb-2011-12-5-r51)

**ADDITIONAL DATA FILE 3: Figures S1-S8**

**Complete Columbian mammoth mitogenome suggests interbreeding with woolly mammoths**

**JACOB ENK,*^,1^ ALISON DEVAULT,^1^ REGIS DEBRUYNE,^1,2^ CHRISTINE E. KING, ^1^ TODD TREANGEN,^3^ DENNIS O’ROURKE,^4^ STEVEN L. SALZBERG,^3^ DANIEL FISHER,^5^ ROSS MACPHEE,^6^ and HENDRIK POINAR*^,1^**

^1^McMaster Ancient DNA Centre, Department of Anthropology, McMaster University, 1280 Main Street West, Hamilton, Ontario L8S 4L9, Canada

^2^Muséum national d'Histoire naturelle, UMR 7206 Eco-anthropologie, Equipe "génétique des populations humaines," 57 rue Cuvier, CP139, 75231 Paris Cedex 05

^3^Center for Bioinformatics and Computational Biology, 3115 Biomolecular Sciences Bldg #296, University of Maryland, College Park, MD 20742

^4^Department of Anthropology, University of Utah, 270 S. 1400 East Room 102, Salt Lake City, UT 84112-0060

^5^Museum of Paleontology and Department of Geological Sciences, University of Michigan, 1109 Geddes Ave. , Ann Arbor, MI 48109-1079

^6^Division of Vertebrate Zoology, American Museum of Natural History, Central Park West @ 79^th^ St, New York, NY 10024

*Corresponding authors:

Jacob Enk ([enkjm@mcmaster.ca](mailto:enkjm@mcmaster.ca)) or Hendrik Poinar ([poinarh@mcmaster.ca](mailto:poinarh@mcmaster.ca))

McMaster Ancient DNA Centre, CNH 524

McMaster University

1280 Main St. West

Hamilton, Ontario L8S 4L9, Canada

P: 1+ 905.525.9140 x26331; F: 1+ 905.522.5993

Keywords: ancient DNA; mammoths; phylogenetics; Pleistocene; North America

**All literature cited refer to those in the main manuscript.**

**FIGURES**

**Fig. S1.** The Huntington mammoth lower left third molar. Figure from [17] used with permission from the *Journal of Paleontology*.


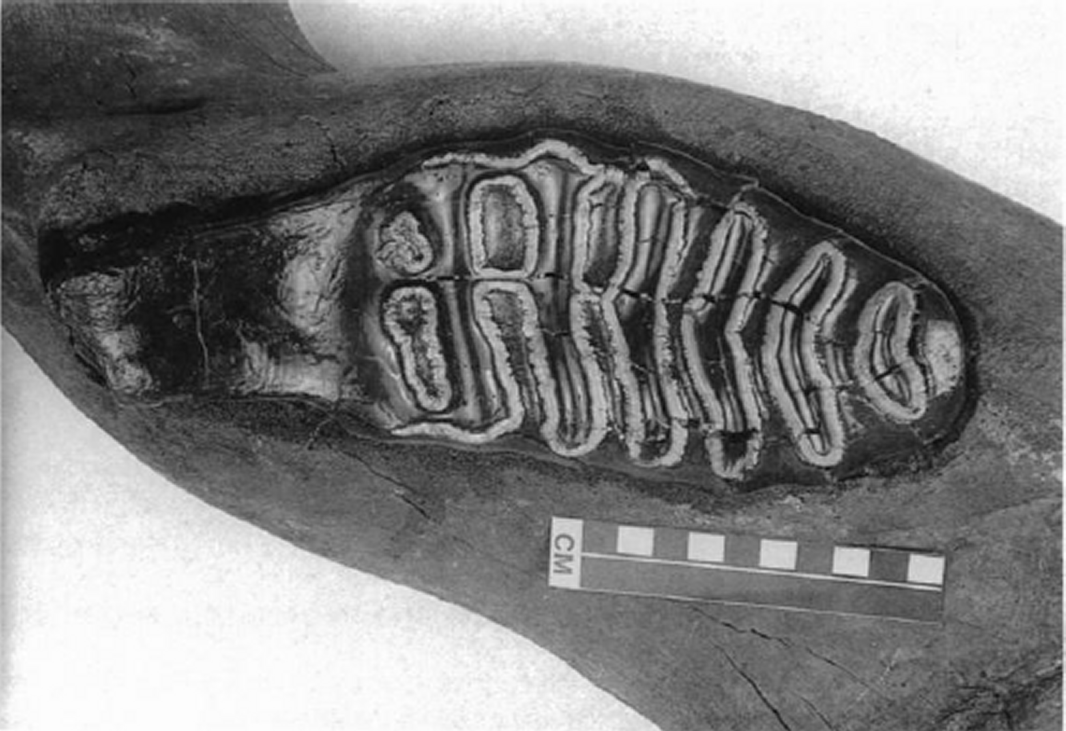


**Fig. S2.** Log-transformed amplicon copy number per milligram of substrate vs. fragment length. Open squares are woolly mammoth samples assayed by [24], with regression lines indicated.


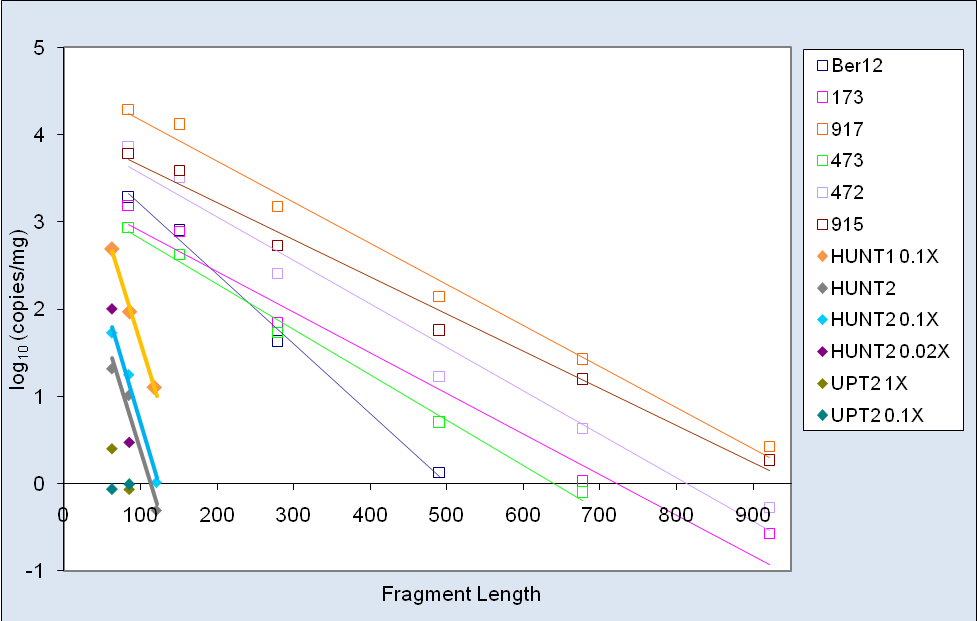


**Fig. S3.** Maximum clade credibility tree from phylogenetic analysis set 1a, with nodal posterior probabilities indicated. Tip names are preceded by their haplotype as determined by [11] and [12].


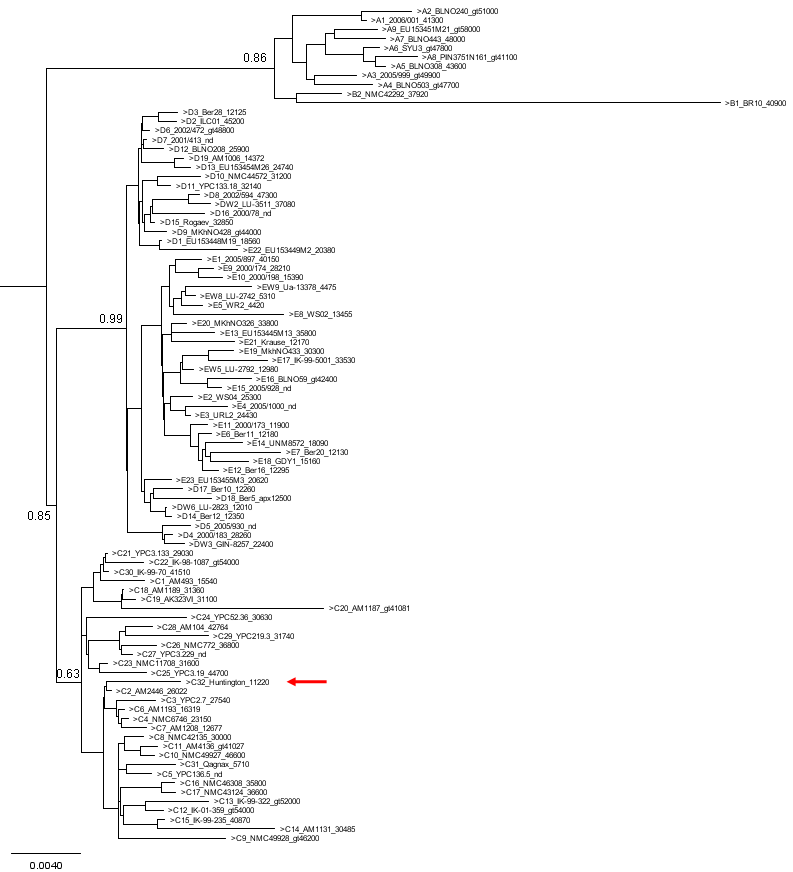


**Fig. S4.** Maximum clade credibility tree from phylogenetic analysis set 1b, with nodal posterior probabilities indicated. Tip names are preceded by their haplotype as determined by [11] and [12].


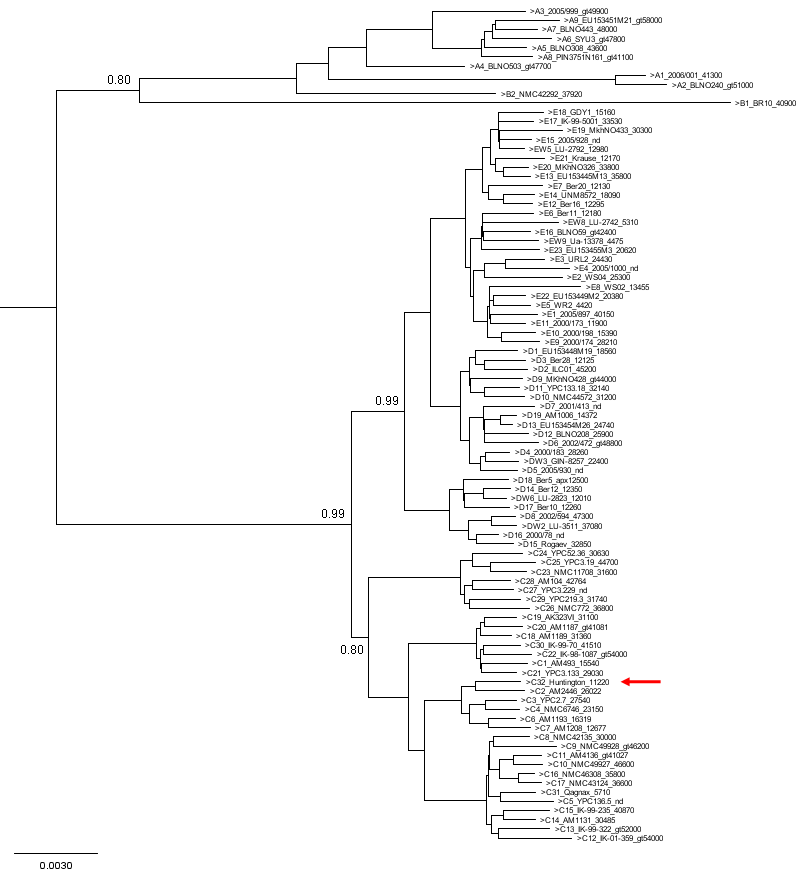


**Fig. S5.** Maximum clade credibility tree from phylogenetic analysis set 2a, with nodal posterior probabilities indicated. Tip names are preceded by their haplotype as determined by [11] and [12].


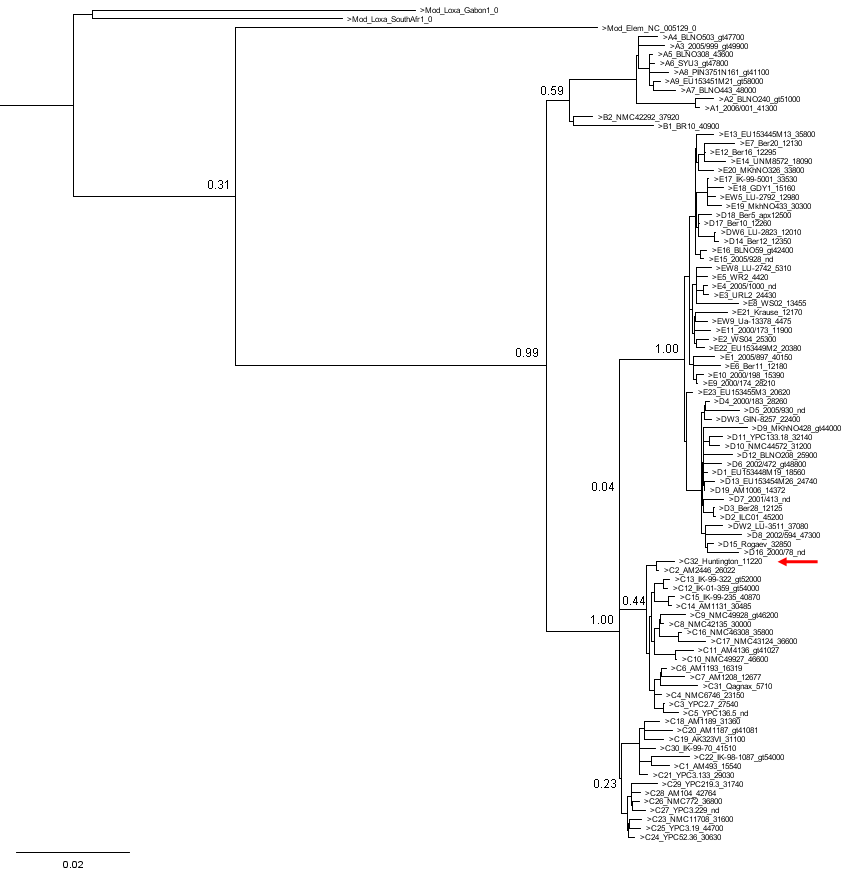


**Fig. S6.** Maximum clade credibility tree from phylogenetic analysis set 2b, with nodal posterior probabilities indicated. Tip names are preceded by their haplotype as determined by [11] and [12].


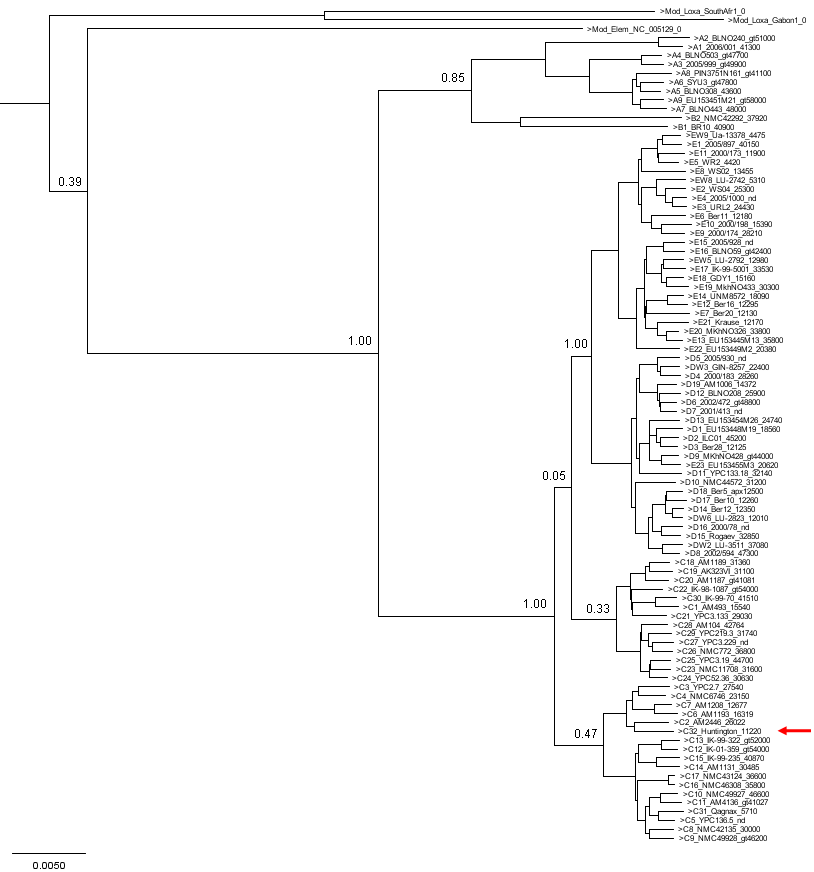


**Fig. S7.** Maximum clade credibility tree from phylogenetic analysis set 4a, with nodal posterior probabilities indicated. Tip names are preceded by their haplotype as determined by [11] and [12].


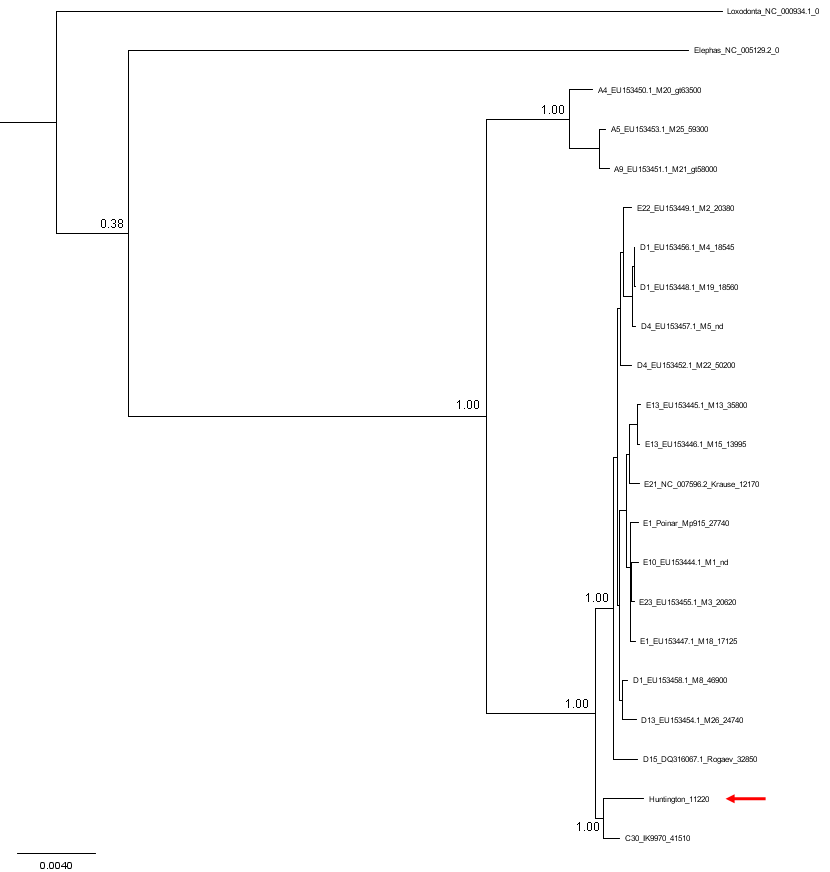


**Fig. S8.** Maximum clade credibility tree from phylogenetic analysis set 4b, with nodal posterior probabilities indicated. Tip names are preceded by their haplotype as determined by [11] and [12].


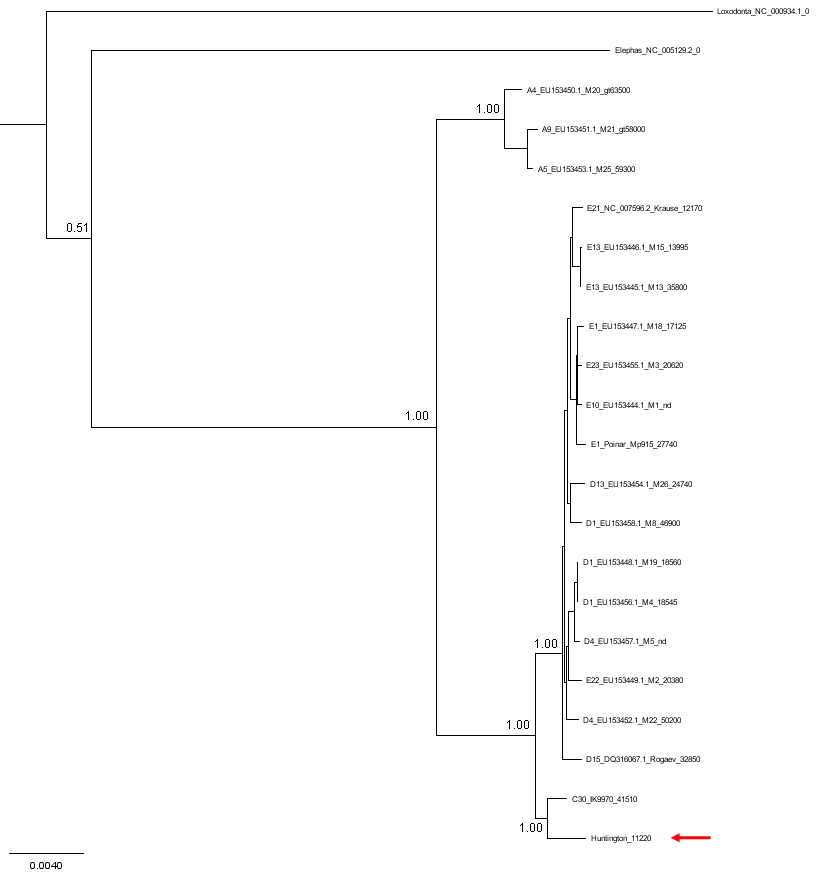

Supplement: Additional File 3 — Additional figures. A collection of figures referred to in the text as Figures S1 to S8. [file gb-2011-12-5-r51-S3.DOC]
